# Subtype-Dependent Transcriptional Coupling Between DNMT1 and E2F-Linked Proliferative Programs in Breast Cancer: A Dual-Cohort Transcriptomic Analysis

**DOI:** 10.7150/jca.134046

**Published:** 2026-08-24

**Authors:** Ping Xiao, Yiqun Xie, Ruijie Niu, Ming Shan, Zhiwu Dong, Yang Shi

**Affiliations:** 1 Department of Breast Surgery, Shanghai Second People's Hospital, Shanghai, China.; 2 Department of Laboratory Medicine, Shanghai Second People's Hospital, Shanghai, China.; 3 Department of Medical Oncology, Shanghai Second People's Hospital, Shanghai, China.

**Keywords:** DNMT1, E2F-linked proliferative programs, RB-E2F axis, breast cancer, transcriptional coupling, public transcriptomics

## Abstract

**Background:**

DNMT1 is a maintenance DNA methyltransferase involved in replication-coupled epigenetic regulation and is frequently expressed in highly proliferative tumors. Although DNMT1 has been linked to RB-E2F-related transcriptional programs, the reproducibility and subtype dependence of this association in breast cancer remain incompletely defined.

**Methods:**

We performed a dual-cohort integrative transcriptomic analysis using TCGA-BRCA as the discovery cohort and METABRIC as the external validation cohort. DNMT1 expression was evaluated across tumor status and subtype annotations, with PAM50 used for all core multivariable and interaction analyses and METABRIC CLAUDIN_SUBTYPE used only for descriptive stratification. DNMT1-associated transcriptional programs were characterized in TCGA-BRCA using differential expression analysis and pathway enrichment. E2F-related transcriptional activity was summarized using an E2F metagene score derived from 122 available genes in the MSigDB Hallmark E2F Targets gene set. Cross-cohort Spearman correlation analyses, multivariable linear models, MKI67-adjusted sensitivity analyses, and likelihood-ratio tests for E2F2 × PAM50 interaction were performed.

**Results:**

DNMT1 expression was elevated in breast tumors and was highest in basal-like tumors. In TCGA-BRCA, high DNMT1 expression was associated with proliferation- and replication-related transcriptional programs, including cell-cycle progression, DNA replication, homologous recombination, and replication stress-related pathways. Across TCGA-BRCA and METABRIC, DNMT1 expression was positively associated with the E2F metagene score, E2F2, E2F3, and MKI67. In multivariable models adjusted for PAM50 subtype and age, the E2F metagene score remained associated with DNMT1 expression in both TCGA-BRCA (standardized β = 0.889, 95% CI 0.837-0.941) and METABRIC (β = 0.578, 95% CI 0.517-0.638). In joint E2F2/E2F3 models, cross-cohort evidence was strongest for E2F2, whereas the E2F3 association was more cohort-dependent and was attenuated in METABRIC. MKI67 adjustment attenuated but did not abolish the E2F2 association in both cohorts. Formal interaction testing supported subtype-dependent heterogeneity in the E2F2-DNMT1 association in both TCGA-BRCA and METABRIC.

**Conclusions:**

DNMT1 shows reproducible, subtype-dependent transcriptional coupling with E2F-linked proliferative programs in breast cancer, with the most consistent cross-cohort evidence observed for the E2F metagene score and E2F2. These findings should be interpreted as hypothesis-generating transcriptomic associations rather than evidence of direct mechanistic causality.

## Introduction

Breast cancer remains the most commonly diagnosed malignancy among women worldwide 1,2. Contemporary clinical overviews also emphasize that breast cancer is a biologically heterogeneous disease requiring subtype-aware interpretation 2,3. Foundational gene-expression studies established intrinsic molecular subtypes of breast cancer, including luminal, HER2-enriched, and basal-like groups 4,5,6. Comprehensive TCGA profiling further confirmed the genomic and transcriptomic heterogeneity of human breast tumors 7. Basal-like tumors frequently overlap with, but are not identical to, triple-negative breast cancer, a heterogeneous clinical entity encompassing multiple molecular subtypes 8,9,10. Additional analyses of triple-negative breast cancer have emphasized biologically distinct subgroup structures and ongoing challenges in subtype-aware interpretation 11,12. Highly proliferative subtypes, including basal-like tumors, are characterized by deregulated cell-cycle control and genomic instability, contributing to aggressive tumor biology 7,13. Clarifying transcriptional programs associated with proliferative states may improve understanding of subtype-specific tumor biology and help prioritize candidate pathways for further investigation.

DNMT1 is the principal maintenance DNA methyltransferase that preserves CpG methylation during DNA replication 14,15,16. Early mechanistic work showed that DNMT1 is targeted to sites of DNA replication in mammalian nuclei 17. The replication-coupled DNMT1/UHRF1 maintenance machinery also involves UHRF1-dependent mechanisms that help connect newly replicated chromatin with maintenance methylation 18,19. Aberrant DNA methylation is widely recognized as an important layer of cancer biology and epigenetic dysregulation 20,21. In breast cancer, DNA methylation has been investigated in relation to tumor biology, risk stratification, and biomarker-oriented research 22,23,24. Breast cancer-specific studies have reported subtype-context differences in DNMT1-related expression and have linked DNMT1 to mammary and cancer stem-cell maintenance, tumorigenesis, and growth/metastasis-related programs 25,26,27. Other breast cancer studies have implicated DNMT1-associated biology in stromal features and organ-specific metastatic programs 28,29. Beyond maintenance methylation, DNMT1 has been implicated in DNA damage response biology and replication fork stability, providing biological precedent for links between DNMT1, genome stability, and replication-associated stress 30,31,32. The RB-E2F axis is a canonical regulator of G1/S transition and cancer-associated cell-cycle deregulation 33,34,35. Cell-cycle transcriptional programs during G1 and S phase coordinate expression of genes required for DNA replication and cell-cycle progression 36. In breast cancer, RB pathway alterations have been discussed in relation to prognosis, subtype context, and therapeutic response biology 37. Earlier mechanistic studies suggested interactions between DNMT1 and E2F-associated regulatory networks, including E2F-responsive elements in the DNMT1 promoter and complex formation involving RB, E2F, and HDAC1 38,39. However, the extent to which DNMT1 expression is associated with specific proliferative E2F branches across large human breast cancer cohorts—and whether such associations differ by intrinsic subtype—remains incompletely characterized.

Here, we performed integrative analyses across TCGA-BRCA and METABRIC to (i) characterize DNMT1 expression across breast cancer subtypes; (ii) define transcriptional programs associated with DNMT1 expression; and (iii) evaluate the association between DNMT1 and RB-E2F-related proliferative programs using pathway activity scoring, multivariable modeling, and subtype interaction analyses. We specifically sought to determine whether DNMT1-E2F coupling showed branch preference and subtype-dependent heterogeneity at the transcriptomic level, rather than to infer direct regulatory causality.

## Materials and Methods

### Data acquisition and preprocessing

TCGA-BRCA RNA-seq count data and clinicopathologic annotations were downloaded from the Genomic Data Commons using TCGAbiolinks version 2.38.0 on May 4, 2026 under GDC project TCGA-BRCA 40,41,42. Primary breast tumor samples with available gene expression data and relevant clinicopathologic annotations were included in the main tumor-based analyses. Where tumor-normal expression comparisons were performed, available TCGA-BRCA normal breast tissue samples were used for descriptive comparison. RNA-seq count data were normalized using edgeR and transformed to log2 counts per million 43. Gene identifiers were mapped to official gene symbols using biomaRt, and duplicated gene symbols were summarized before downstream analysis 44.

For external validation, processed METABRIC expression data and clinical annotations were downloaded from cBioPortal on May 4, 2026 using the study identifier brca_metabric. METABRIC was used as an external breast cancer validation cohort based on its genomic and transcriptomic characterization of large breast tumor series 45,46. cBioPortal was used for access to processed expression and clinical annotation data 47,48. Expression and clinical data were harmonized by sample identifiers.

Because different analyses required different combinations of expression variables, subtype annotations, age, and proliferation-marker availability, samples were retained using analysis-specific complete-case sets. Tumor-normal comparisons used all available TCGA-BRCA samples for that comparison; subtype-stratified expression analyses used samples with available subtype annotation; DNMT1-stratified differential expression and enrichment analyses used TCGA-BRCA primary tumors with available DNMT1 expression; correlation analyses required DNMT1, the E2F metagene score, E2F2, E2F3, and MKI67; multivariable models required DNMT1 expression, the relevant E2F-related variable, PAM50 subtype, and age; and MKI67-adjusted sensitivity models additionally required MKI67. Samples lacking a variable required for a specific analysis were excluded only from that analysis. Analysis-specific sample sizes are reported in the corresponding Results sections, figures, and supplementary model-output tables.

**Figure 1:**
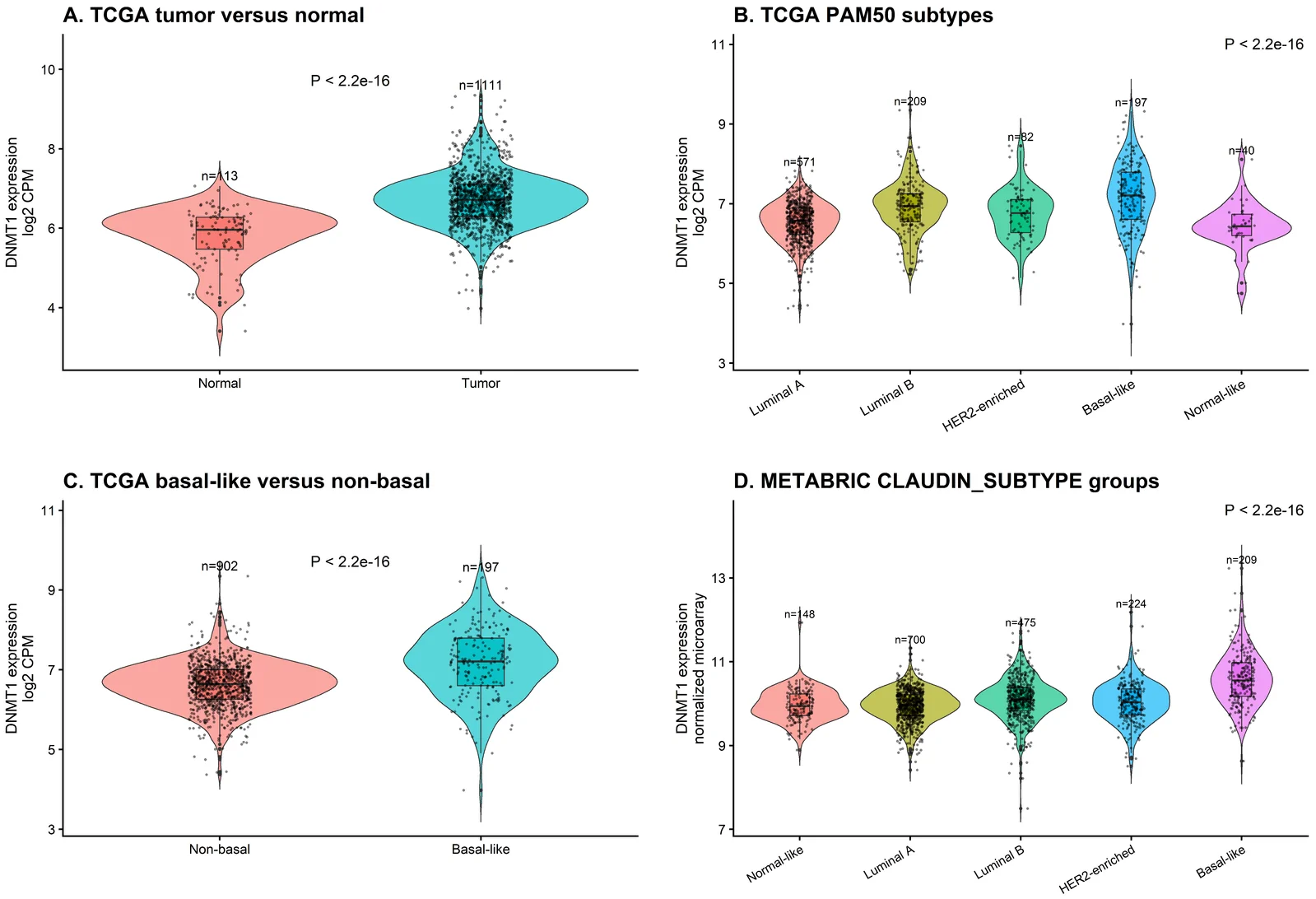
** DNMT1 expression across breast cancer tissues and molecular subtype groups. (A)** DNMT1 expression in TCGA-BRCA non-tumor breast tissue and primary breast tumors. **(B)** DNMT1 expression across PAM50 intrinsic subtypes in TCGA-BRCA. **(C)** DNMT1 expression in basal-like versus non-basal tumors in TCGA-BRCA. **(D)** DNMT1 expression across METABRIC subtype groups based on the available CLAUDIN_SUBTYPE annotation, used for descriptive stratification only. Box plots show the median and interquartile range, with violin plots showing the distribution of expression values. Sample sizes are indicated above each group. P values were calculated using Wilcoxon rank-sum tests for two-group comparisons and Kruskal-Wallis tests for multi-group comparisons, as appropriate. TCGA-BRCA expression is shown as log2 CPM, whereas METABRIC expression is shown as normalized microarray expression; expression scales should not be directly compared across cohorts. PAM50 subtype annotation was used for TCGA-BRCA subtype-stratified analyses and for all core subtype-adjusted and interaction models in both cohorts. In METABRIC, CLAUDIN_SUBTYPE annotation was used only for descriptive stratification in panel D and was not used in the core multivariable or interaction models.

PAM50 intrinsic subtype was used as the primary subtype variable for all core multivariable and interaction analyses in both TCGA-BRCA and METABRIC 6. In METABRIC, the available CLAUDIN_SUBTYPE annotation was used only for descriptive DNMT1 expression stratification in Figure 1D and was not included in the core regression or interaction models 49.

### Differential expression and enrichment analyses

Within TCGA-BRCA, primary tumors were divided into DNMT1-high and DNMT1-low groups using the cohort-specific median DNMT1 expression as the cutoff. Differential expression analysis comparing DNMT1-high versus DNMT1-low tumors was performed using the limma-voom workflow 50,51. Positive log2 fold-change values indicate higher expression in DNMT1-high tumors.

**Figure 2:**
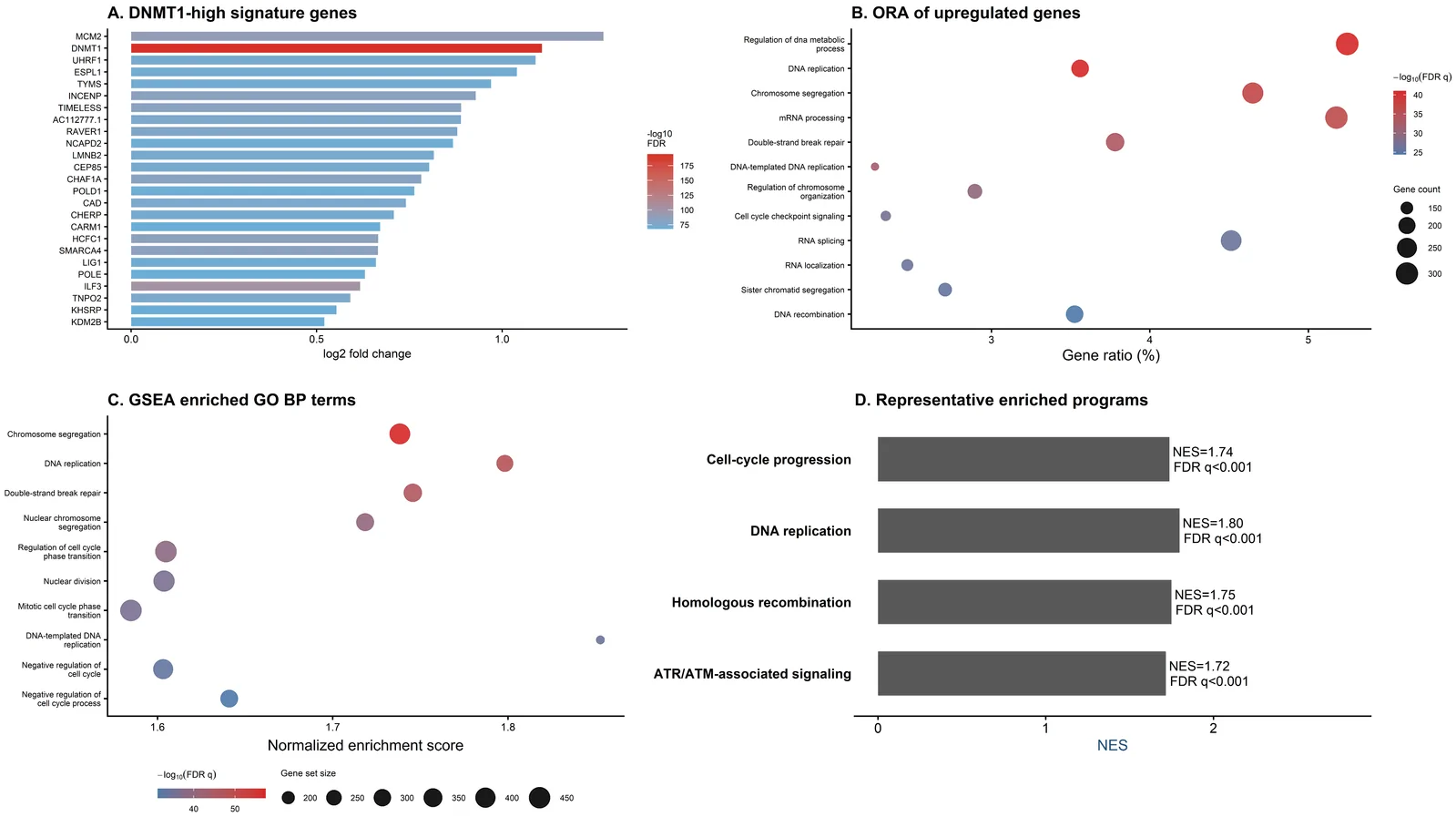
** DNMT1-associated transcriptional programs in TCGA-BRCA. (A)** Differential expression analysis comparing TCGA-BRCA tumors with high versus low DNMT1 expression, defined using the cohort-specific median DNMT1 expression cutoff. **(B)** Over-representation analysis of significantly upregulated genes in DNMT1-high tumors. **(C)** Gene set enrichment analysis of pre-ranked differential expression results highlighting proliferation- and replication-associated pathways. **(D)** Summary of representative enriched pathways related to cell-cycle progression, DNA replication, homologous recombination, and ATR/ATM-associated signaling. For the principal GSEA results discussed in the text, normalized enrichment scores and FDR q values are shown. These analyses were used to characterize DNMT1-associated transcriptional programs in the TCGA-BRCA discovery cohort.

For over-representation analysis, significantly upregulated genes were defined as genes with a positive log2 fold change and a Benjamini-Hochberg adjusted P value < 0.05 in DNMT1-high versus DNMT1-low tumors. No additional absolute log2 fold-change threshold was imposed. Over-representation analysis was conducted using clusterProfiler, with the expressed and mapped genes retained after preprocessing used as the background gene universe. Gene Ontology Biological Process and Reactome pathway resources were considered where reported 52,53,54. Gene set enrichment analysis was performed using a pre-ranked gene list according to the GSEA framework 55. Genes were ranked by the moderated t statistic from the limma-voom differential expression analysis, with positive values indicating higher expression in DNMT1-high tumors. Pre-ranked GSEA was performed using MSigDB gene-set resources, with Gene Ontology Biological Process terms used for the enrichment analyses shown in Figure 2 and Figure S2. The MSigDB Hallmark E2F Targets gene set was used separately for E2F metagene construction as described below 56,57. Enrichment results with FDR q < 0.05 were considered statistically significant, and normalized enrichment scores and FDR q values were reported for the principal pathways discussed in the manuscript.

### E2F-related metagene scoring

E2F-related transcriptional activity was summarized using genes from the MSigDB Hallmark E2F Targets gene set that were available in both TCGA-BRCA and METABRIC 57. In the final analysis, 122 E2F target genes were retained for metagene construction. For each cohort, expression values for each retained E2F target gene were standardized across samples using z-score transformation. The E2F metagene score for each sample was then calculated as the mean of the standardized expression values across the retained E2F target genes.

This metagene score was used as a transcriptomic-level summary of E2F-related proliferative activity rather than as evidence of direct E2F transcriptional regulation of DNMT1.

### Correlation and multivariable modeling

Associations between DNMT1 expression and E2F-related variables were first evaluated using Spearman correlation analyses. The variables assessed included the E2F metagene score, E2F2, E2F3, and MKI67. Spearman correlation coefficients, two-sided nominal P values, Benjamini-Hochberg adjusted q values where applicable, and sample sizes were reported for each cohort.

For regression-based analyses, continuous variables were standardized using z-score transformation to facilitate comparison of effect sizes. Multivariable linear regression models were fitted using analysis-specific complete cases, with standardized DNMT1 expression as the outcome. The primary models evaluated the association between DNMT1 and the E2F metagene score after adjustment for PAM50 subtype and age. Additional models included E2F2 and E2F3 simultaneously, with adjustment for PAM50 subtype and age.

Because DNMT1 and E2F-related transcriptional programs are closely linked to proliferative state, sensitivity analyses were performed by additionally adjusting for MKI67 as a representative proliferation marker in breast cancer 58,59. These analyses were intended to evaluate whether the observed DNMT1-E2F associations were fully reducible to MKI67, rather than to establish independence from proliferative state.

### Subtype interaction analysis

Subtype-dependent heterogeneity in the E2F2-DNMT1 association was assessed using formal interaction testing. For each cohort, a main-effects model including E2F2, PAM50 subtype, and age was compared with a corresponding model including an E2F2 × PAM50 interaction term. Likelihood-ratio tests were used to compare model fit between the two models. Luminal A was used as the reference subtype in all PAM50-based regression and interaction models.

### Statistical analysis

All statistical analyses were performed in R. Unless otherwise specified, tests were two-sided, and P < 0.05 was considered statistically significant. Where multiple testing was relevant, Benjamini-Hochberg FDR correction was applied 60. Unless explicitly described as FDR-adjusted q values, reported P values are nominal two-sided P values. Model results are reported as standardized β coefficients with 95% confidence intervals, nominal P values, adjusted R² values, and sample sizes.

### Use of artificial intelligence tools

During manuscript preparation and revision, OpenAI ChatGPT was used as an assistive tool for English-language refinement, manuscript organization, and code troubleshooting. All analysis code was executed by the authors, and all data processing, statistical outputs, figures, references, interpretations, and final manuscript text were independently reviewed and verified by the authors. The AI tool was not listed as an author and did not make autonomous scientific or editorial decisions.

## Results

The overall dual-cohort analysis workflow is summarized in Figure S1.

### DNMT1 expression is elevated in breast tumors and highest in basal-like disease

In TCGA-BRCA, DNMT1 expression was significantly higher in breast tumors than in normal breast tissue and differed across PAM50 intrinsic subtypes, with the highest expression observed in basal-like tumors (Fig. 1A-C). In METABRIC, DNMT1 expression was examined descriptively across the available CLAUDIN_SUBTYPE groups shown in Figure 1D, with higher expression observed in basal-like tumors (Fig. 1D). The METABRIC CLAUDIN_SUBTYPE annotation was used only for descriptive stratification and was not included in the core PAM50-based regression or interaction analyses.

### Tumors with high DNMT1 expression are enriched for proliferative and replication-associated transcriptional programs

Differential expression analysis in TCGA-BRCA comparing tumors with high versus low DNMT1 expression identified DNMT1-associated transcriptional programs enriched for cell-cycle progression, DNA replication, homologous recombination, and replication stress-related pathways (Fig. 2). Over-representation analysis and GSEA consistently highlighted proliferative and replication-associated pathways, including Cell Cycle, DNA Replication, Homologous Recombination, and ATR/ATM-related signaling. For the principal enrichment results discussed in the manuscript, normalized enrichment scores and FDR q values are shown in Fig. 2 and Figure S2. These findings indicate that high DNMT1 expression is associated with a proliferative transcriptional state, providing the rationale for subsequent analyses of E2F-related variables and proliferation-adjusted sensitivity models.

### DNMT1 expression is positively associated with E2F-related transcriptional variables across independent cohorts

**Figure 3:**
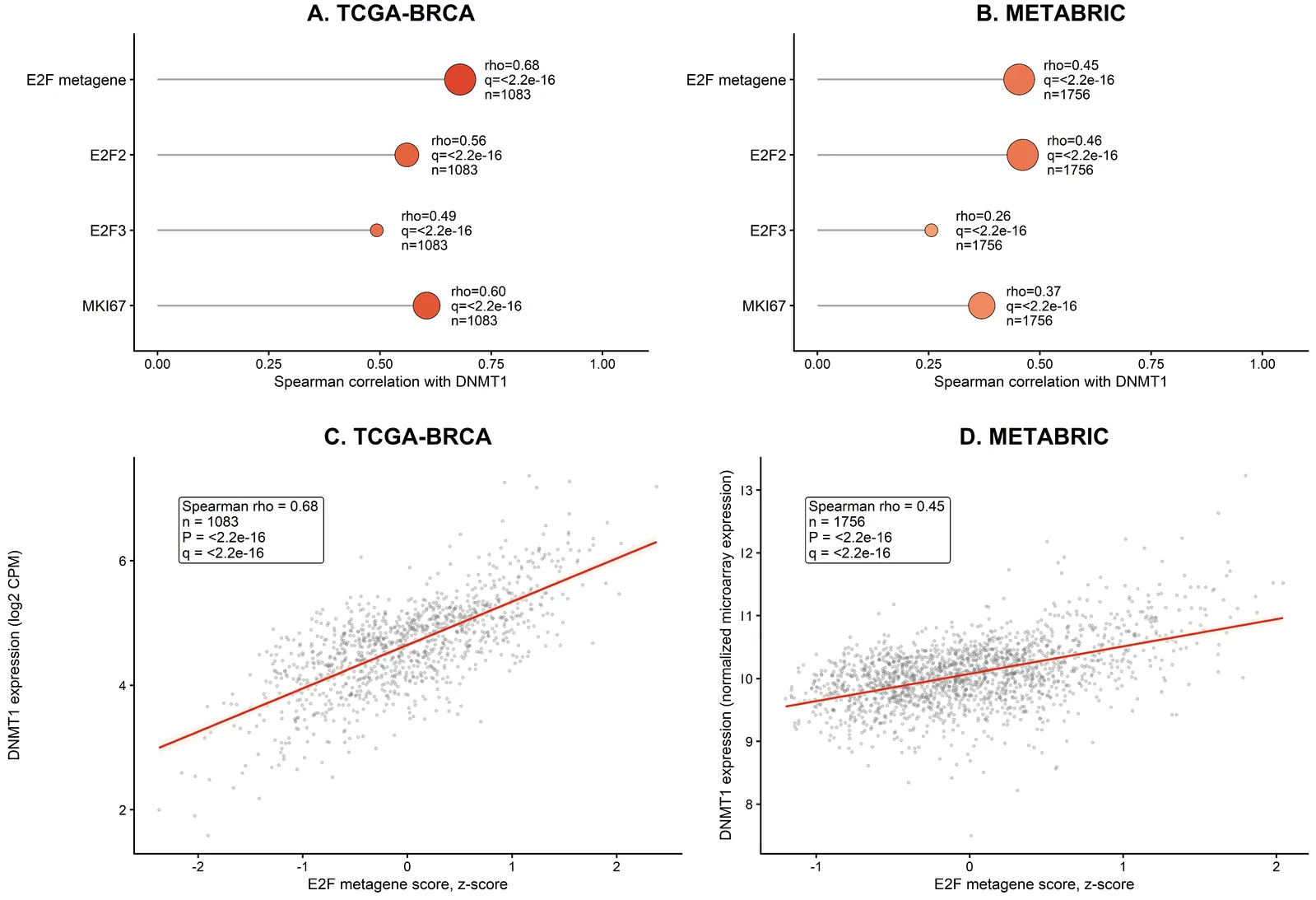
** Cross-cohort associations between DNMT1 expression and E2F-related transcriptional variables. (A)** Spearman correlation summary showing the associations between DNMT1 expression and the E2F metagene score, E2F2, E2F3, and MKI67 in TCGA-BRCA. **(B)** Spearman correlation summary showing the same associations in METABRIC. **(C)** Scatter plot showing the association between DNMT1 expression and the cohort-standardized E2F metagene score in TCGA-BRCA. **(D)** Scatter plot showing the association between DNMT1 expression and the cohort-standardized E2F metagene score in METABRIC.TCGA-BRCA DNMT1 expression is shown as log2 CPM, whereas METABRIC DNMT1 expression is shown as normalized microarray expression; these expression scales should not be directly compared across cohorts. Spearman correlation coefficients, sample sizes, nominal P values, and Benjamini-Hochberg adjusted q values are shown where applicable. Lines in panels C and D indicate fitted linear trends with confidence bands for visualization. These analyses evaluate transcriptomic-level associations and do not imply direct regulatory relationships. The corresponding lower-triangular pairwise correlation matrices are provided in Figure S3.

Given the enrichment of proliferation- and replication-associated programs in DNMT1-high tumors, we next evaluated the association between DNMT1 expression and E2F-related transcriptional variables across TCGA-BRCA and METABRIC. In TCGA-BRCA, DNMT1 expression correlated positively with the E2F metagene score (Spearman ρ = 0.680, P = 6.48 × 10⁻^148^), E2F2 (ρ = 0.560, P = 1.46 × 10⁻^90^), E2F3 (ρ = 0.493, P = 2.35 × 10⁻⁶⁷), and MKI67 (ρ = 0.605, P = 4.65 × 10⁻^109^; n = 1,083; Fig. 3A).

A similar positive direction was observed in METABRIC, although the effect sizes were smaller. DNMT1 expression correlated with the E2F metagene score (ρ = 0.454, P = 8.19 × 10⁻^90^), E2F2 (ρ = 0.461, P = 3.54 × 10⁻^93^), E2F3 (ρ = 0.256, P = 1.08 × 10⁻^27^), and MKI67 (ρ = 0.369, P = 7.33 × 10⁻^58^; n = 1,756; Fig. 3B). These cross-cohort results support a reproducible transcriptomic association between DNMT1 expression and E2F-linked proliferative programs, with the strongest consistency among E2F-related variables observed for the E2F metagene score and E2F2. Pairwise lower-triangular Spearman correlation matrices for DNMT1, the E2F metagene score, E2F2, E2F3, and MKI67 are provided in Figure S3.

### E2F-related metagene activity remains associated with DNMT1 expression after adjustment for PAM50 subtype and age

**Figure 4:**
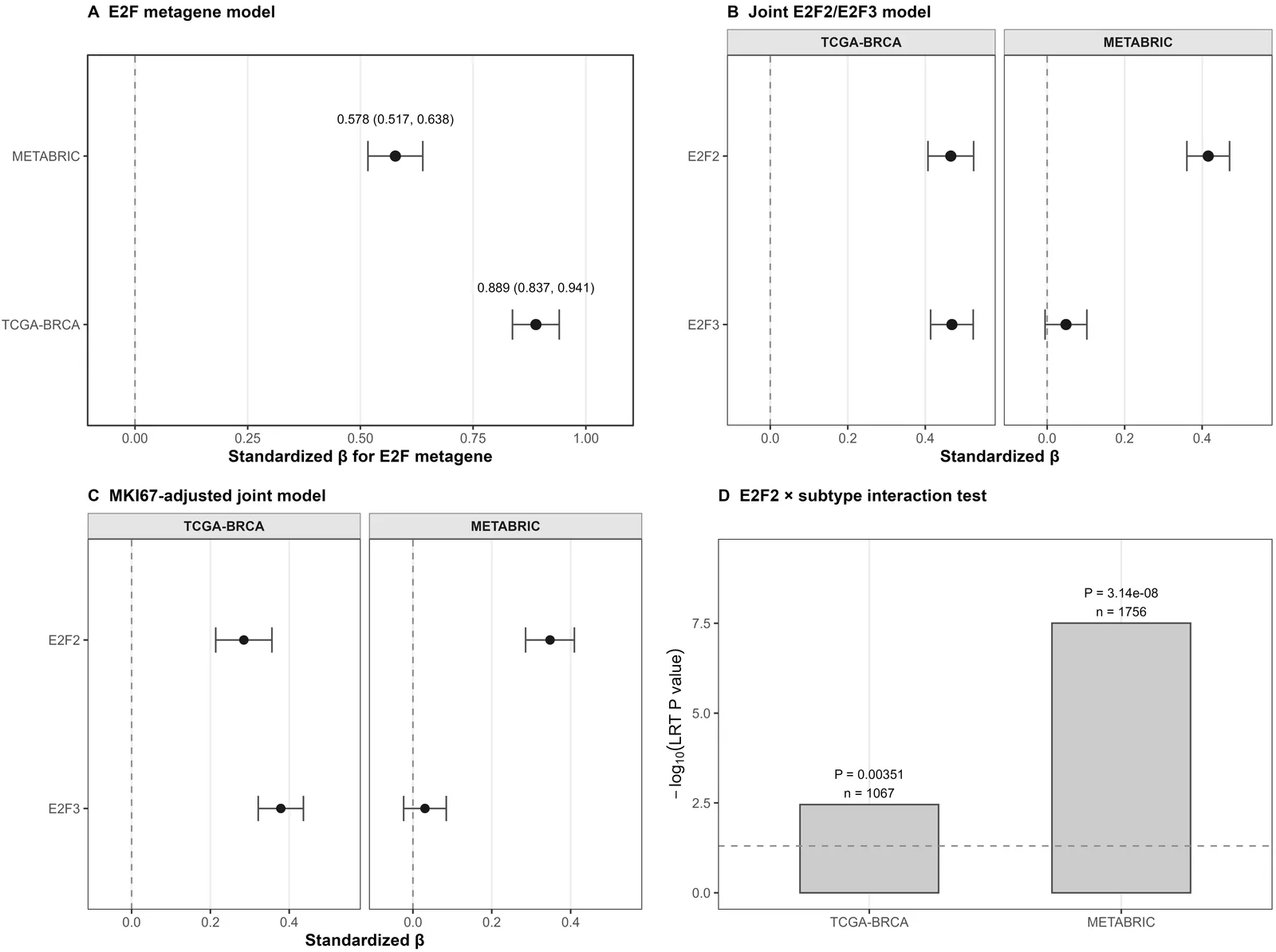
** Cross-cohort multivariable and subtype-interaction analyses of the DNMT1-E2F association. (A)** Multivariable linear models evaluating the association between the E2F metagene score and standardized DNMT1 expression after adjustment for age and PAM50 subtype annotation in TCGA-BRCA and METABRIC. **(B)** Joint E2F2/E2F3 models evaluating the associations of individual E2F variables with standardized DNMT1 expression after adjustment for age and PAM50 subtype annotation in both cohorts. **(C)** MKI67-adjusted joint E2F2/E2F3 models evaluating the extent to which E2F2 and E2F3 associations were attenuated after additional adjustment for a representative proliferation marker. **(D)** E2F2 × PAM50 subtype interaction analyses using likelihood-ratio tests comparing models with and without an E2F2 × subtype interaction term within each cohort. All multivariable and E2F2 × subtype interaction models shown in Figure 4 used PAM50 subtype annotation in both cohorts, with Luminal A as the reference subtype. METABRIC CLAUDIN_SUBTYPE annotation was used only for the descriptive analysis shown in Figure 1D. E2F2 was selected for formal subtype-interaction visualization because it showed the most consistent cross-cohort association with DNMT1 among individual E2F variables. Detailed MKI67-adjusted sensitivity-model outputs are provided in Supplementary Table S1. Extended regression and subtype-interaction visualizations are provided in Figure S4. Detailed subtype-specific E2F2 slope estimates, interaction contrast estimates relative to Luminal A, 95% confidence intervals, nominal P values, Benjamini-Hochberg adjusted q values, global likelihood-ratio test statistics, and sample sizes are provided in Supplementary Table S2. Standardized β coefficients and 95% confidence intervals are shown in panels A-C. Panel D shows -log10-transformed likelihood-ratio test P values, with the dashed horizontal line indicating -log10(0.05). These analyses evaluate subtype-dependent heterogeneity in the E2F2-DNMT1 association and do not establish a subtype-specific regulatory mechanism.

We next fitted multivariable linear models to determine whether E2F-related transcriptional activity remained associated with DNMT1 expression after adjustment for PAM50 subtype and age. In TCGA-BRCA, the E2F metagene score was positively associated with DNMT1 expression after adjustment for these covariates (standardized β = 0.889, 95% CI 0.837-0.941, P = 7.32 × 10⁻^170^; adjusted R² = 0.558; n = 1,067; Fig. 4A). This association was also observed in METABRIC (standardized β = 0.578, 95% CI 0.517-0.638, P = 9.45 × 10⁻^71^; adjusted R² = 0.290; n = 1,756; Fig. 4A).

When E2F2 and E2F3 were included simultaneously in the same multivariable model, both genes were positively associated with DNMT1 expression in TCGA-BRCA. The standardize β was 0.466 for E2F2 (95% CI 0.407-0.525, P = 4.78 × 10⁻⁴⁹) and 0.469 for E2F3 (95% CI 0.414-0.524, P = 5.60 × 10⁻^56^; adjusted R² = 0.458; n = 1,067; Fig. 4B). In METABRIC, E2F2 remained positively associated with DNMT1 expression in the joint model (standardized β = 0.416, 95% CI 0.361-0.471, P = 9.91 × 10⁻⁴⁷), whereas the E2F3 association was attenuated and did not reach statistical significance (standardized β = 0.049, 95% CI -0.005 to 0.103, P = 0.077; adjusted R² = 0.258; n = 1,756; Fig. 4B). Thus, the cross-cohort evidence was most consistent for E2F metagene activity and E2F2, whereas the E2F3 association appeared more cohort-dependent.

### MKI67-adjusted sensitivity analyses indicate partial contribution of proliferative state

Because DNMT1, E2F-related transcriptional activity, and MKI67 are all linked to proliferative state, we performed MKI67-adjusted sensitivity analyses. In TCGA-BRCA, additional adjustment for MKI67 attenuated the E2F2 association in the joint E2F2/E2F3 model from β = 0.466 to β = 0.285, but the association remained statistically significant (95% CI 0.214-0.356, P = 1.13 × 10⁻^14^). The E2F3 association was also attenuated from β = 0.469 to β = 0.379 and remained statistically significant (95% CI 0.321-0.436, P = 9.19 × 10⁻^36^). The adjusted R² increased from 0.458 to 0.491 after inclusion of MKI67 (n = 1,067; Fig. 4C).

In METABRIC, E2F2 similarly remained associated with DNMT1 expression after MKI67 adjustment, although the effect size decreased from β = 0.416 to β = 0.348 (95% CI 0.286-0.409, P = 1.90 × 10⁻^27^). By contrast, E2F3 remained non-significant in the joint model after MKI67 adjustment (β = 0.030, 95% CI -0.024 to 0.085, P = 0.270). The adjusted R² increased modestly from 0.258 to 0.267 (n = 1,756; Fig. 4C). These findings indicate that proliferative state contributes to the observed DNMT1-E2F associations. However, the E2F2 association was not fully reducible to MKI67 alone, whereas the E2F3-related signal was less stable across cohorts and model specifications. Detailed MKI67-adjusted model terms, confidence intervals, nominal P values, q values, model-fit indices, and sample sizes are provided in Supplementary Table S1.

### The E2F2-DNMT1 association shows subtype-dependent heterogeneity

To formally evaluate whether the E2F2-DNMT1 association differed across intrinsic subtypes, we compared models with and without an E2F2 × PAM50 interaction term using likelihood-ratio tests. In TCGA-BRCA, the interaction model improved model fit compared with the corresponding main-effects model (χ² = 15.66, df = 4, likelihood-ratio test P = 0.0035; n = 1,067; Fig. 4D). This pattern was also observed in METABRIC, where the E2F2 × PAM50 interaction was significant by likelihood-ratio testing (χ² = 40.68, df = 4, P = 3.14 × 10⁻⁸; n = 1,756; Fig. 4D).

These results support subtype-dependent heterogeneity in the strength of the E2F2-DNMT1 association across breast cancer intrinsic subtypes. They should be interpreted as evidence of context-dependent transcriptomic coupling rather than as proof of a subtype-specific regulatory mechanism. Extended regression and subtype-interaction visualizations are provided in Figure S4. Detailed subtype-specific E2F2 slope estimates, interaction contrast estimates relative to Luminal A, 95% confidence intervals, nominal P values, Benjamini-Hochberg adjusted q values, global likelihood-ratio test statistics, and sample sizes are provided in Supplementary Table S2.

## Discussion

In this dual-cohort integrative transcriptomic analysis of TCGA-BRCA and METABRIC, we identified reproducible associations between DNMT1 expression and E2F-linked proliferative transcriptional programs in breast cancer. Across cohorts, DNMT1 expression was highest in basal-like tumors and was positively associated with proliferation- and replication-related signatures. These findings are consistent with the established replication-coupled role of DNMT1 in maintenance DNA methylation and genome stability 14,16,17, as well as with breast cancer-specific studies linking DNMT1 dysregulation to molecular subtype context, tumorigenesis, and aggressive phenotypes 25-27. Additional studies have implicated DNMT1-associated programs in stromal biology and metastatic progression, although these observations should not be interpreted as evidence of causality in the present transcriptomic analysis 28,29. Importantly, this study was designed to characterize cohort-level transcriptomic associations rather than to establish direct regulatory causality.

A central observation of this analysis is that the association between DNMT1 and E2F-related activity was reproducible but not uniform across intrinsic subtypes. Formal interaction testing supported subtype-dependent heterogeneity in the E2F2-DNMT1 association in both TCGA-BRCA and METABRIC. This heterogeneity may reflect differences in baseline proliferative state, lineage context, chromatin organization, and the magnitude of RB-E2F program activation across breast cancer subtypes. Accordingly, stronger associations within particular subtypes should be interpreted as evidence of context-dependent transcriptional coordination rather than proof of a subtype-specific regulatory mechanism.

The cross-cohort evidence was strongest for the E2F metagene score and E2F2. In multivariable models adjusted for PAM50 subtype and age, E2F metagene activity remained positively associated with DNMT1 expression in both cohorts. When E2F2 and E2F3 were modeled simultaneously, E2F2 showed a consistent positive association with DNMT1 in both TCGA-BRCA and METABRIC. By contrast, the association with E2F3 was evident in TCGA-BRCA but was attenuated and did not reach statistical significance in the METABRIC joint E2F2/E2F3 model. The E2F3-related findings should therefore be interpreted cautiously. Rather than supporting a uniform E2F2/3 pattern across cohorts, the revised results support a more conservative interpretation: DNMT1 shows reproducible transcriptional coupling with E2F-linked proliferative programs, with the most consistent individual-gene evidence observed for E2F2.

Because DNMT1, E2F-related programs, and MKI67 are all linked to cell-cycle activity, confounding by the general proliferative state is an important interpretive consideration. MKI67 has been widely used and standardized as a proliferation marker in breast cancer assessment, although its analytical and clinical interpretation requires caution 58,59. In sensitivity analyses adjusted for MKI67, the associations between DNMT1 and E2F-related variables were attenuated, indicating that the general proliferative state contributes to the observed relationships. Nevertheless, the association between E2F2 and DNMT1 remained statistically significant after MKI67 adjustment in both cohorts, suggesting that this relationship was not fully captured by MKI67 alone. At the same time, it should not be described as independent of proliferation, because DNMT1 expression and E2F activity remain biologically and transcriptionally intertwined with proliferative tumor states.

The present data also do not resolve regulatory directionality. One plausible interpretation is that E2F-enriched proliferative states contribute to increased DNMT1 expression in highly cycling tumors. This interpretation is consistent with the role of RB-E2F activity in coordinating the expression of genes involved in G1/S transition and DNA replication 33,34,36. Conversely, because DNMT1 is a maintenance DNA methyltransferase, increased DNMT1 expression may influence the transcriptional landscape of selected E2F-responsive genes or their upstream regulators through methylation-dependent or chromatin-associated mechanisms. Earlier studies identifying E2F-responsive elements in the DNMT1 promoter and regulatory complexes involving RB, E2F, and HDAC1 provide biological precedent for potentially bidirectional relationships 38,39. However, these possibilities remain inferential and cannot be established using bulk transcriptomic data alone.

Beyond intrinsic molecular subtype, the observed DNMT1-E2F associations should also be interpreted in the context of cellular heterogeneity within breast tumors. Recent single-cell and multi-omics studies have identified a GNA15-overexpressing malignant-cell cluster associated with malignancy-related pathways and inferior survival in triple-negative breast cancer, as well as an SPP1-positive tumor-associated macrophage state associated with poor prognosis in triple-negative disease 61,62. In estrogen receptor-positive breast cancer, integrated single-cell and spatial transcriptomic analyses identified COL1A2-positive/MMP1-positive and COL1A2-positive/MMP1-negative cancer-associated fibroblast populations associated with unfavorable outcomes and extensive crosstalk with tumor-associated macrophages 63. Collectively, these studies illustrate that clinically relevant transcriptomic programs may arise from distinct malignant, immune, and stromal compartments and may vary according to cellular and spatial context. Accordingly, the DNMT1-E2F associations identified in the present bulk-transcriptomic analysis cannot be assigned to a specific cell population or spatial niche. Future studies integrating single-cell and spatial transcriptomics with methylation profiling and functional perturbation will be required to determine whether DNMT1-E2F2 coupling is concentrated within particular malignant-cell states or is influenced by interactions with immune and stromal components of the tumor microenvironment.

Several limitations should be emphasized. First, this study was based on public bulk transcriptomic cohorts and therefore cannot establish direct mechanistic causality, physical regulatory interactions, or functional dependency in breast cancer models. Second, no matched protein-level, methylation-level, ChIP-based, or perturbational validation was performed. Third, residual confounding by proliferative state remains possible, even after adjustment for MKI67 as a representative proliferation marker. Fourth, bulk transcriptomic data cannot resolve the malignant, immune, or stromal cellular origin of the observed associations. Fifth, TCGA-BRCA and METABRIC differ in expression platforms, preprocessing frameworks, and the completeness of clinical annotations, which may influence effect-size estimates and cross-cohort comparability. Finally, differences in subtype annotation and sample size across intrinsic subtypes may affect the precision and interpretation of the interaction analyses. The present findings should therefore be regarded as hypothesis-generating transcriptomic associations.

In summary, DNMT1 showed reproducible and subtype-dependent associations with E2F-linked proliferative transcriptional programs across TCGA-BRCA and METABRIC, with E2F2 providing the most consistent individual-gene signal across cohorts. These findings provide a subtype-aware framework for subsequent mechanistic and cell type-resolved investigation, rather than evidence of direct transcriptional regulation or functional dependency.

## Conclusion

In summary, this dual-cohort transcriptomic analysis of TCGA-BRCA and METABRIC identified reproducible, subtype-dependent associations between DNMT1 expression and E2F-linked proliferative transcriptional programs in breast cancer. The most consistent cross-cohort evidence was observed for the E2F metagene score and E2F2, whereas the E2F3 association appeared more cohort-dependent. MKI67-adjusted sensitivity analyses indicated that proliferative state contributes to these associations, but the DNMT1-E2F2 relationship was not fully reducible to MKI67 alone. These findings support a context-dependent pattern of DNMT1-E2F transcriptional coupling at the cohort level, particularly in highly proliferative tumor states, but do not establish direct mechanistic causality, functional dependency, or therapeutic tractability. The present study should therefore be interpreted as hypothesis-generating and as a framework for prioritizing subtype-aware DNMT1-E2F relationships for further functional validation.

## Supplementary Material

Supplementary figures and tables.

## Data Availability

TCGA-BRCA data are publicly available from the Genomic Data Commons under project TCGA-BRCA. METABRIC processed expression and clinical annotation data are publicly available from cBioPortal under the study identifier brca_metabric. The processed analysis matrices, R scripts, statistical model outputs, and figure-generation code required to reproduce the reported results are publicly available in the Open Science Framework (OSF) at https://osf.io/4smvu/.
