# Supplementary material for: Subtype-Dependent Transcriptional Coupling Between DNMT1 and E2F-Linked Proliferative Programs in Breast Cancer: A Dual-Cohort Transcriptomic Analysis
Source: J Cancer. 2026 Aug 24;17(9):1547–57. doi: 10.7150/jca.134046 (PMC13573300; doi:10.7150/jca.134046)
Supplement: Supplementary file 1 [file jcav17p1547s1.pdf]

Supplementary Materials

Subtype-Dependent Transcriptional Coupling Between DNMT1 and E2F-Linked Proliferative Programs in Breast Cancer: A Dual-Cohort Transcriptomic Analysis

Ping Xiao1#*, Yiqun Xie1#, Ruijie Niu1*, Ming Shan1, Zhiwu Dong2, Yang Shi3

Contents

- Figure S1 - Dual-cohort transcriptomic analysis workflow
- Figure S2 - Extended GSEA of DNMT1-associated programs
- Figure S3 - Cross-cohort Spearman correlation matrices
- Figure S4 - Extended regression and subtype-interaction analyses
- Supplementary Table S1 - MKI67-adjusted sensitivity analyses
- Supplementary Table S2 - Subtype-specific E2F2-DNMT1 interaction analyses

Figures are reproduced from the supplied 1200 dpi source files. Supplementary tables are presented in paginated, human-readable form; the original XLSX files remain the authoritative machine-readable versions.

FIGURE S1

Overview of the dual-cohort transcriptomic analysis workflow.

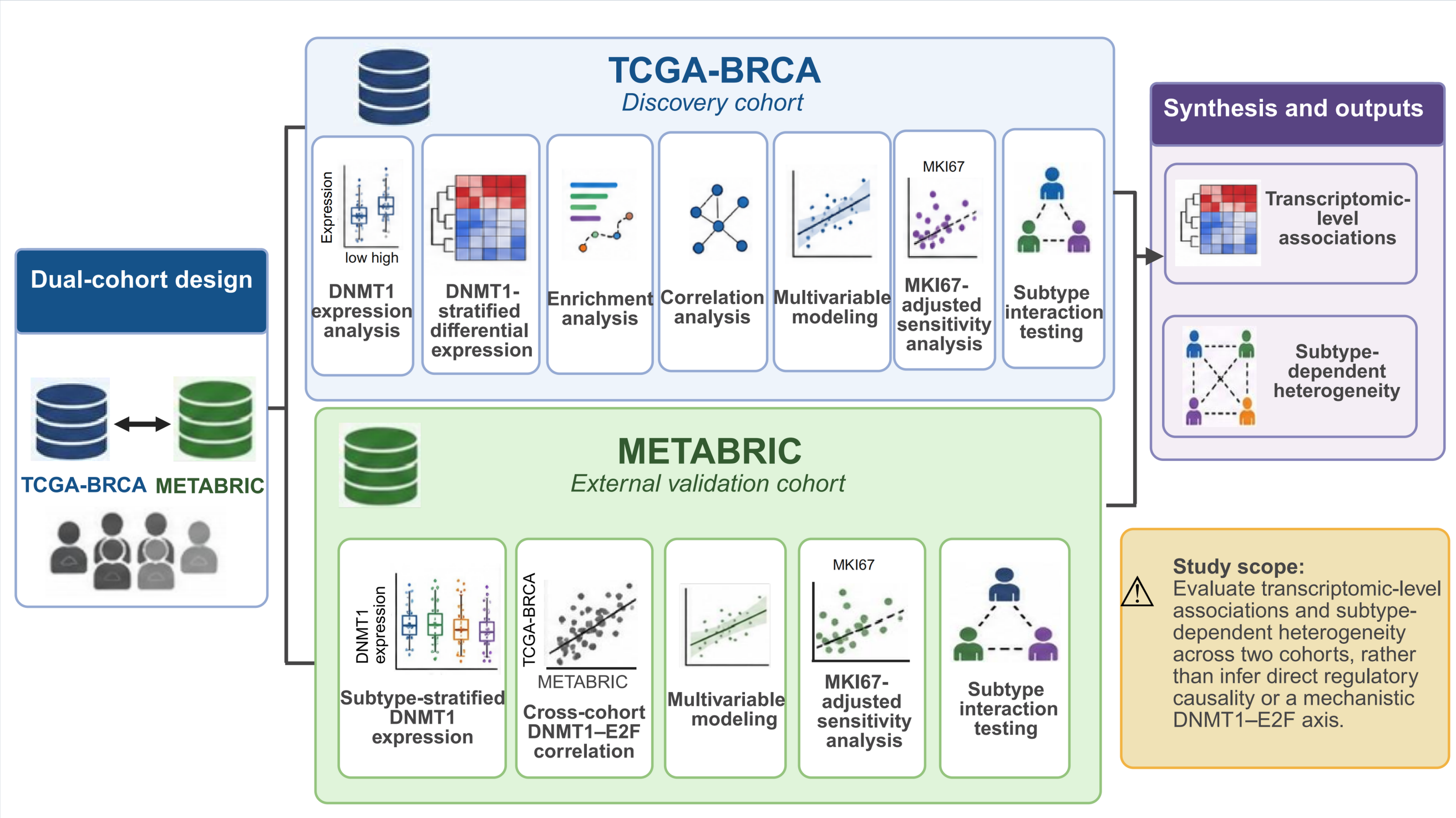

Legend

Schematic overview of the revised dual-cohort analysis design. TCGA-BRCA was used as the discovery cohort for DNMT1 expression analysis, DNMT1-stratified differential expression, enrichment analysis, correlation analysis, multivariable modeling, MKI67-adjusted sensitivity analysis, and subtype interaction testing. METABRIC was used as an external validation cohort for subtype-stratified DNMT1 expression, cross-cohort DNMT1–E2F correlation analysis, multivariable modeling, MKI67-adjusted sensitivity analysis, and subtype interaction testing. The workflow was designed to evaluate transcriptomic-level associations and subtype-dependent heterogeneity across two cohorts, rather than to infer direct regulatory causality or a mechanistic DNMT1–E2F axis.

FIGURE S2

Extended GSEA of DNMT1-associated transcriptional programs in TCGA-BRCA.

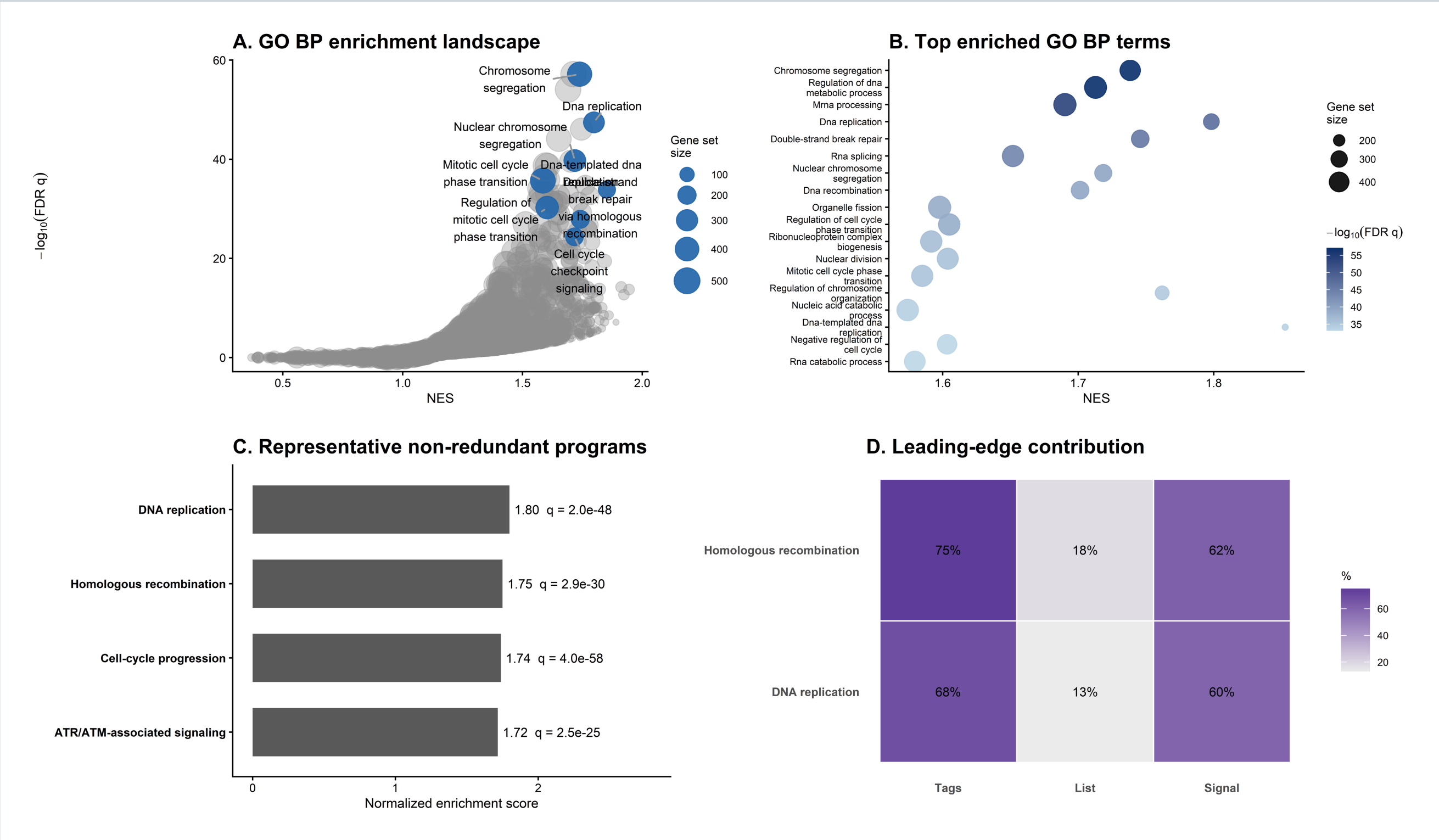

Legend

Extended gene set enrichment analysis of TCGA-BRCA tumors comparing DNMT1-high and DNMT1-low groups defined by the cohort-specific median DNMT1 expression cutoff. (A) Global enrichment landscape of positively enriched GO Biological Process terms in DNMT1-high tumors, plotted by normalized enrichment score and FDR q value. Highlighted points indicate representative non-redundant transcriptional programs. (B) Top enriched GO Biological Process terms ranked by FDR q value, with point size indicating gene set size. (C) Representative proliferation-, replication-, cell-cycle checkpoint-, homologous recombination-, and DNA damage response-associated programs selected from the full GSEA output. (D) Leading-edge metrics for the representative programs, including tag fraction, ranked-list position, and signal strength. Positive NES indicates enrichment in DNMT1-high tumors. These analyses characterize DNMT1-associated transcriptional programs in the TCGA-BRCA discovery cohort.

FIGURE S3

Lower-triangular Spearman correlation matrices of DNMT1 and E2F-related variables in TCGA-BRCA and METABRIC.

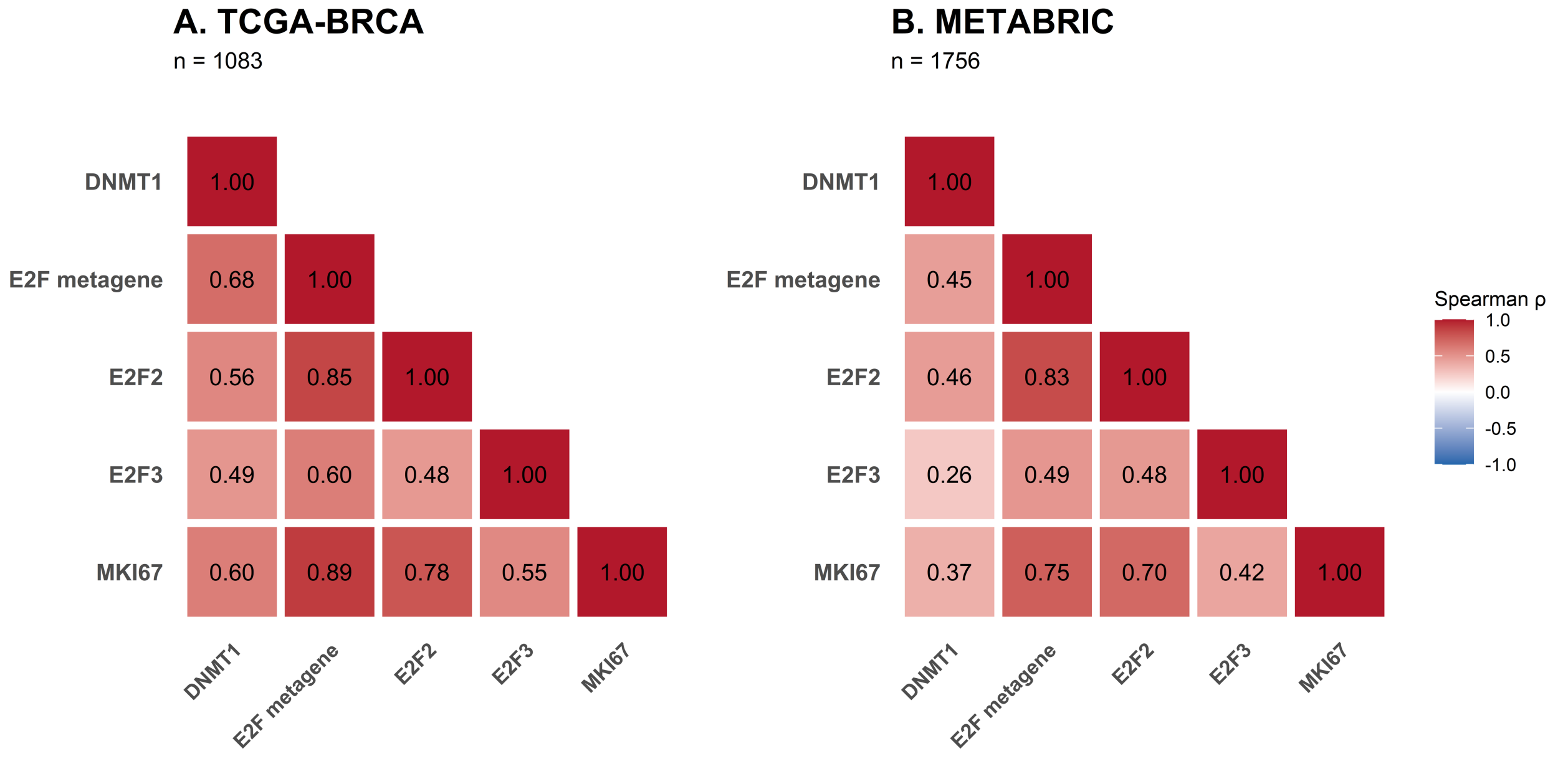

Only the lower triangle is displayed to avoid redundancy. Diagonal cells indicate self-correlation values of ρ = 1.00, and upper-triangular cells are intentionally left blank.

Legend

Spearman correlation matrices showing pairwise associations among DNMT1 expression, the E2F metagene score, E2F2, E2F3, and MKI67 in TCGA-BRCA and METABRIC. DNMT1 showed positive correlations with the E2F metagene score, E2F2, E2F3, and MKI67 in both cohorts. The E2F3 association was weaker in METABRIC than in TCGA-BRCA, whereas the E2F metagene score and E2F2 showed more consistent cross-cohort associations. Spearman ρ values are shown in the heatmap cells. These analyses were used to characterize transcriptomic-level associations and do not imply direct regulatory relationships.

FIGURE S4

Extended regression and subtype-interaction analyses of DNMT1–E2F associations in TCGA-BRCA and METABRIC.

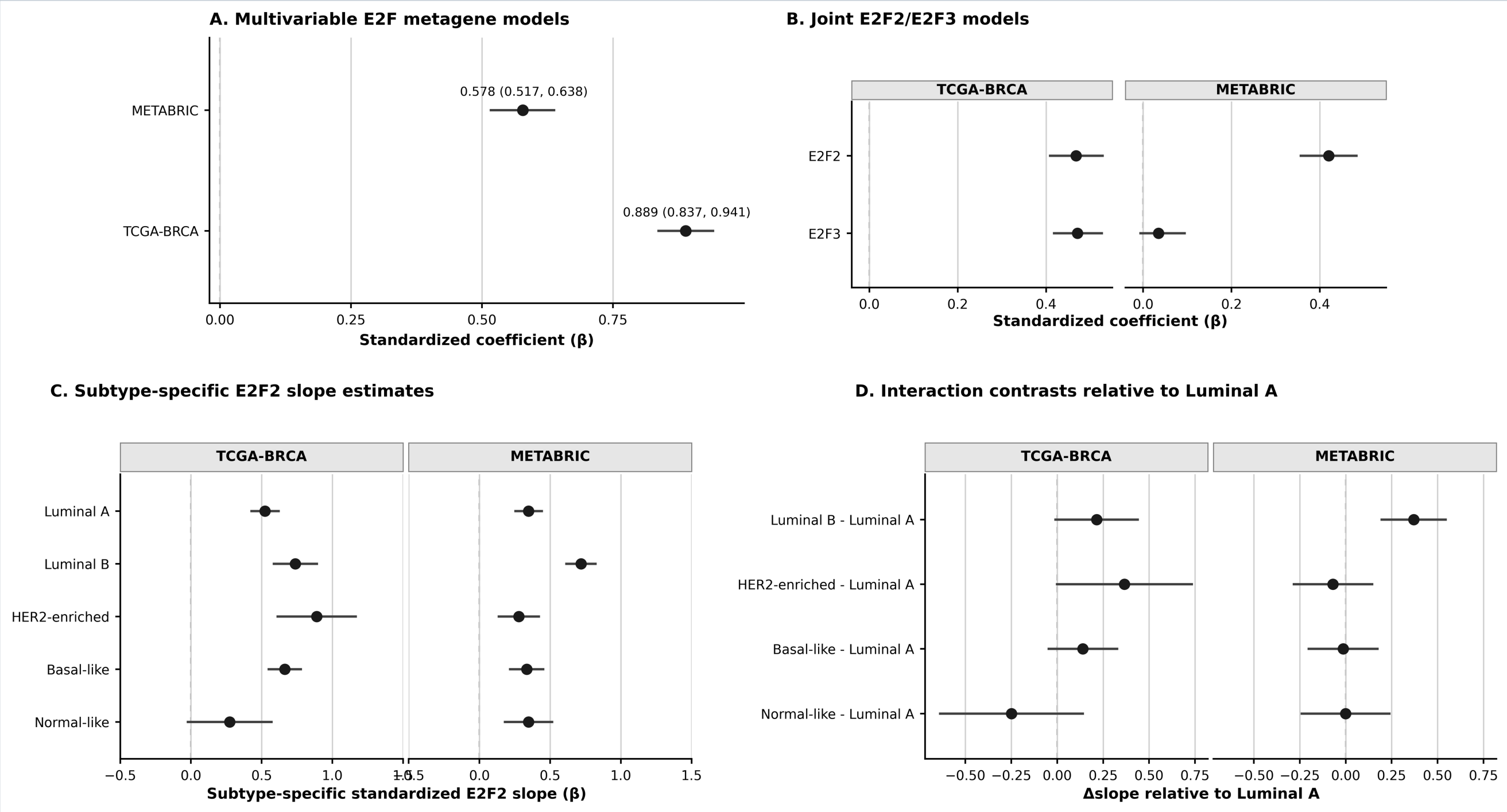

Legend

(A) Multivariable E2F metagene models evaluating associations with standardized DNMT1 expression in TCGA-BRCA and METABRIC after adjustment for age and PAM50 subtype. (B) Joint E2F2/E2F3 models showing standardized coefficients for E2F2 and E2F3 in each cohort after adjustment for age and PAM50 subtype. (C) Model-derived subtype-specific E2F2 slope estimates from PAM50-based E2F2 × subtype interaction models. (D) Interaction contrast estimates comparing each non-reference PAM50 subtype with Luminal A. Points indicate standardized slope estimates or Δslope estimates, as appropriate, and horizontal bars indicate 95% confidence intervals. Subtype-interaction analyses used PAM50 subtype annotation in both cohorts, with Luminal A as the reference subtype. The METABRIC CLAUDIN_SUBTYPE groups shown descriptively in Figure 1D were not included as separate interaction categories in the core PAM50-based models. The slope panel visualizes the estimated strength of the E2F2–DNMT1 association within each subtype, whereas the contrast panel visualizes differences in slope relative to Luminal A. Detailed subtype-specific slope estimates, interaction contrasts, exact P values, q values, likelihood-ratio test statistics, and sample sizes for the subtype-interaction analyses shown in panels C and D are provided in Supplementary Table S2. These analyses evaluate subtype-dependent heterogeneity in transcriptomic association and do not establish a subtype-specific regulatory mechanism.

SUPPLEMENTARY TABLE S1

Definitions and analysis notes

Supplementary Table S1 provides the MKI67-adjusted sensitivity-model terms, including standardized estimates, standard errors, 95% confidence intervals, nominal P values, Benjamini–Hochberg adjusted q values where applicable, adjusted R^2 values, AIC, BIC, and sample sizes. These analyses were used to assess the extent to which DNMT1–E2F associations were attenuated after adjustment for proliferative state, rather than to establish independence from proliferation.

Item	Description
Outcome	Standardized DNMT1 expression
Cohorts	TCGA-BRCA and METABRIC
Base models	E2F metagene, E2F2, E2F3, or joint E2F2/E2F3 terms adjusted for subtype and age
Sensitivity models	Base models additionally adjusted for standardized MKI67
Subtype reference	Luminal A where PAM50-based subtype terms were modeled
Proliferation marker	MKI67
PCNA	Not included
Interpretation	Used to assess attenuation after proliferation adjustment, not independence from proliferation

SUPPLEMENTARY TABLE S1

Focal terms summary - coefficients and significance

Core MKI67-adjusted focal terms; numerical values are reproduced from the supplied workbook.

cohort	model	outcome	term	estimate	std error	statistic	p value	q value BH
TCGA-BRCA	E2F2_plus_MKI67	DNMT1_z	E2F2_z	0.2816	0.0391	7.2003	1.138e-12	4.551e-12
TCGA-BRCA	E2F2_plus_MKI67	DNMT1_z	MKI67_z	0.4911	0.0374	13.1240	1.445e-36	1.156e-35
TCGA-BRCA	E2F3_plus_MKI67	DNMT1_z	E2F3_z	0.3772	0.0300	12.5540	8.444e-34	3.378e-33
TCGA-BRCA	E2F3_plus_MKI67	DNMT1_z	MKI67_z	0.4871	0.0308	15.7980	1.215e-50	9.721e-50
TCGA-BRCA	E2F_metagene_plus_MKI67	DNMT1_z	E2F_metagene_z	0.9788	0.0473	20.7029	3.220e-80	2.576e-79
TCGA-BRCA	E2F_metagene_plus_MKI67	DNMT1_z	MKI67_z	-0.1016	0.0445	-2.2810	0.0227	0.0303
TCGA-BRCA	E2F2_E2F3_joint_plus_MKI67	DNMT1_z	E2F2_z	0.2849	0.0364	7.8363	1.126e-14	2.533e-14
TCGA-BRCA	E2F2_E2F3_joint_plus_MKI67	DNMT1_z	E2F3_z	0.3788	0.0292	12.9608	9.195e-36	8.275e-35
TCGA-BRCA	E2F2_E2F3_joint_plus_MKI67	DNMT1_z	MKI67_z	0.3114	0.0374	8.3166	2.761e-16	8.283e-16
METABRIC	E2F2_plus_MKI67	DNMT1_z	E2F2_z	0.3544	0.0309	11.4698	2.050e-29	1.640e-28
METABRIC	E2F2_plus_MKI67	DNMT1_z	MKI67_z	0.1426	0.0292	4.8881	1.111e-06	2.963e-06
METABRIC	E2F3_plus_MKI67	DNMT1_z	E2F3_z	0.0893	0.0280	3.1891	0.0015	0.0039
METABRIC	E2F3_plus_MKI67	DNMT1_z	MKI67_z	0.2881	0.0270	10.6568	9.806e-26	7.845e-25
METABRIC	E2F_metagene_plus_MKI67	DNMT1_z	E2F_metagene_z	0.5274	0.0377	14.0009	2.705e-42	2.164e-41
METABRIC	E2F_metagene_plus_MKI67	DNMT1_z	MKI67_z	0.0707	0.0302	2.3429	0.0192	0.0308
METABRIC	E2F2_E2F3_joint_plus_MKI67	DNMT1_z	E2F2_z	0.3477	0.0315	11.0416	1.902e-27	1.712e-26
METABRIC	E2F2_E2F3_joint_plus_MKI67	DNMT1_z	E2F3_z	0.0305	0.0276	1.1032	0.2701	0.3220
METABRIC	E2F2_E2F3_joint_plus_MKI67	DNMT1_z	MKI67_z	0.1381	0.0295	4.6847	3.020e-06	9.060e-06

SUPPLEMENTARY TABLE S1

Focal terms summary - confidence intervals and model fit

The cohort, model, and term columns are repeated to keep this wide table readable.

cohort	model	term	conf low	conf high	n	adj r squared	AIC	BIC	error
TCGA-BRCA	E2F2_plus_MKI67	E2F2_z	0.2049	0.3584	1,067	0.4110	2460.77	2505.53	
TCGA-BRCA	E2F2_plus_MKI67	MKI67_z	0.4177	0.5645	1,067	0.4110	2460.77	2505.53	
TCGA-BRCA	E2F3_plus_MKI67	E2F3_z	0.3182	0.4361	1,067	0.4622	2363.74	2408.49	
TCGA-BRCA	E2F3_plus_MKI67	MKI67_z	0.4266	0.5476	1,067	0.4622	2363.74	2408.49	
TCGA-BRCA	E2F_metagene_plus_MKI67	E2F_metagene_z	0.8860	1.0715	1,067	0.5602	2149.16	2193.91	
TCGA-BRCA	E2F_metagene_plus_MKI67	MKI67_z	-0.1889	-0.0142	1,067	0.5602	2149.16	2193.91	
TCGA-BRCA	E2F2_E2F3_joint_plus_MKI67	E2F2_z	0.2135	0.3562	1,067	0.4912	2305.54	2355.27	
TCGA-BRCA	E2F2_E2F3_joint_plus_MKI67	E2F3_z	0.3214	0.4361	1,067	0.4912	2305.54	2355.27	
TCGA-BRCA	E2F2_E2F3_joint_plus_MKI67	MKI67_z	0.2379	0.3848	1,067	0.4912	2305.54	2355.27	
METABRIC	E2F2_plus_MKI67	E2F2_z	0.2938	0.4150	1,756	0.2670	4447.76	4497.00	
METABRIC	E2F2_plus_MKI67	MKI67_z	0.0854	0.1999	1,756	0.2670	4447.76	4497.00	
METABRIC	E2F3_plus_MKI67	E2F3_z	0.0344	0.1442	1,756	0.2164	4564.99	4614.23	
METABRIC	E2F3_plus_MKI67	MKI67_z	0.2351	0.3412	1,756	0.2164	4564.99	4614.23	
METABRIC	E2F_metagene_plus_MKI67	E2F_metagene_z	0.4535	0.6013	1,756	0.2914	4388.54	4437.77	
METABRIC	E2F_metagene_plus_MKI67	MKI67_z	0.0115	0.1298	1,756	0.2914	4388.54	4437.77	
METABRIC	E2F2_E2F3_joint_plus_MKI67	E2F2_z	0.2859	0.4094	1,756	0.2671	4448.54	4503.24	
METABRIC	E2F2_E2F3_joint_plus_MKI67	E2F3_z	-0.0237	0.0846	1,756	0.2671	4448.54	4503.24	
METABRIC	E2F2_E2F3_joint_plus_MKI67	MKI67_z	0.0803	0.1959	1,756	0.2671	4448.54	4503.24	

SUPPLEMENTARY TABLE S1

Full model outputs - coefficients and significance

Part 1 of the full output table. q value BH denotes the Benjamini-Hochberg adjusted q value where applicable.

cohort	model	outcome	term	estimate	std error	statistic	p value	q value BH
TCGA-BRCA	E2F2_plus_MKI67	DNMT1_z	(Intercept)	0.1887	0.0364	5.1879	2.547e-07	5.094e-07
TCGA-BRCA	E2F2_plus_MKI67	DNMT1_z	E2F2_z	0.2816	0.0391	7.2003	1.138e-12	4.551e-12
TCGA-BRCA	E2F2_plus_MKI67	DNMT1_z	MKI67_z	0.4911	0.0374	13.1240	1.445e-36	1.156e-35
TCGA-BRCA	E2F2_plus_MKI67	DNMT1_z	age_z	-0.0025	0.0238	-0.1051	0.9163	0.9163
TCGA-BRCA	E2F2_plus_MKI67	DNMT1_z	PAM50Luminal B	-0.3182	0.0700	-4.5420	6.212e-06	8.283e-06
TCGA-BRCA	E2F2_plus_MKI67	DNMT1_z	PAM50HER2-enriched	-0.6342	0.0972	-6.5234	1.063e-10	2.835e-10
TCGA-BRCA	E2F2_plus_MKI67	DNMT1_z	PAM50Basal-like	-0.3773	0.0793	-4.7586	2.220e-06	3.552e-06
TCGA-BRCA	E2F2_plus_MKI67	DNMT1_z	PAM50Normal-like	-0.1515	0.1257	-1.2056	0.2282	0.2609
TCGA-BRCA	E2F3_plus_MKI67	DNMT1_z	(Intercept)	0.1642	0.0339	4.8515	1.409e-06	2.255e-06
TCGA-BRCA	E2F3_plus_MKI67	DNMT1_z	E2F3_z	0.3772	0.0300	12.5540	8.444e-34	3.378e-33
TCGA-BRCA	E2F3_plus_MKI67	DNMT1_z	MKI67_z	0.4871	0.0308	15.7980	1.215e-50	9.721e-50
TCGA-BRCA	E2F3_plus_MKI67	DNMT1_z	age_z	0.0151	0.0228	0.6603	0.5092	0.5092
TCGA-BRCA	E2F3_plus_MKI67	DNMT1_z	PAM50Luminal B	-0.1727	0.0660	-2.6157	0.0090	0.0120
TCGA-BRCA	E2F3_plus_MKI67	DNMT1_z	PAM50HER2-enriched	-0.5064	0.0922	-5.4944	4.911e-08	9.821e-08
TCGA-BRCA	E2F3_plus_MKI67	DNMT1_z	PAM50Basal-like	-0.4749	0.0745	-6.3746	2.734e-10	7.290e-10
TCGA-BRCA	E2F3_plus_MKI67	DNMT1_z	PAM50Normal-like	-0.1055	0.1199	-0.8793	0.3794	0.4336
TCGA-BRCA	E2F_metagene_plus_MKI67	DNMT1_z	(Intercept)	0.2627	0.0312	8.4198	1.213e-16	2.427e-16
TCGA-BRCA	E2F_metagene_plus_MKI67	DNMT1_z	E2F_metagene_z	0.9788	0.0473	20.7029	3.220e-80	2.576e-79
TCGA-BRCA	E2F_metagene_plus_MKI67	DNMT1_z	MKI67_z	-0.1016	0.0445	-2.2810	0.0227	0.0303
TCGA-BRCA	E2F_metagene_plus_MKI67	DNMT1_z	age_z	0.0040	0.0206	0.1943	0.8460	0.8460
TCGA-BRCA	E2F_metagene_plus_MKI67	DNMT1_z	PAM50Luminal B	-0.5654	0.0618	-9.1549	2.732e-19	1.093e-18
TCGA-BRCA	E2F_metagene_plus_MKI67	DNMT1_z	PAM50HER2-enriched	-0.5650	0.0833	-6.7818	1.969e-11	3.151e-11
TCGA-BRCA	E2F_metagene_plus_MKI67	DNMT1_z	PAM50Basal-like	-0.5964	0.0669	-8.9172	2.055e-18	5.480e-18
TCGA-BRCA	E2F_metagene_plus_MKI67	DNMT1_z	PAM50Normal-like	0.0525	0.1087	0.4830	0.6292	0.7190
TCGA-BRCA	E2F2_E2F3_joint_plus_MKI67	DNMT1_z	(Intercept)	0.2287	0.0339	6.7396	2.606e-11	4.690e-11
TCGA-BRCA	E2F2_E2F3_joint_plus_MKI67	DNMT1_z	E2F2_z	0.2849	0.0364	7.8363	1.126e-14	2.533e-14
TCGA-BRCA	E2F2_E2F3_joint_plus_MKI67	DNMT1_z	E2F3_z	0.3788	0.0292	12.9608	9.195e-36	8.275e-35
TCGA-BRCA	E2F2_E2F3_joint_plus_MKI67	DNMT1_z	MKI67_z	0.3114	0.0374	8.3166	2.761e-16	8.283e-16
TCGA-BRCA	E2F2_E2F3_joint_plus_MKI67	DNMT1_z	age_z	0.0254	0.0222	1.1426	0.2534	0.2534
TCGA-BRCA	E2F2_E2F3_joint_plus_MKI67	DNMT1_z	PAM50Luminal B	-0.2624	0.0652	-4.0218	6.186e-05	7.953e-05
TCGA-BRCA	E2F2_E2F3_joint_plus_MKI67	DNMT1_z	PAM50HER2-enriched	-0.5978	0.0904	-6.6125	5.984e-11	8.976e-11
TCGA-BRCA	E2F2_E2F3_joint_plus_MKI67	DNMT1_z	PAM50Basal-like	-0.6926	0.0776	-8.9242	1.940e-18	8.732e-18
TCGA-BRCA	E2F2_E2F3_joint_plus_MKI67	DNMT1_z	PAM50Normal-like	-0.1505	0.1168	-1.2889	0.1977	0.2224

SUPPLEMENTARY TABLE S1

Full model outputs - coefficients and significance (continued)

Part 1 of the full output table. q value BH denotes the Benjamini-Hochberg adjusted q value where applicable.

cohort	model	outcome	term	estimate	std error	statistic	p value	q value BH
METABRIC	E2F2_plus_MKI67	DNMT1_z	(Intercept)	0.0364	0.0368	0.9891	0.3227	0.3688
METABRIC	E2F2_plus_MKI67	DNMT1_z	E2F2_z	0.3544	0.0309	11.4698	2.050e-29	1.640e-28
METABRIC	E2F2_plus_MKI67	DNMT1_z	MKI67_z	0.1426	0.0292	4.8881	1.111e-06	2.963e-06
METABRIC	E2F2_plus_MKI67	DNMT1_z	age_z	-0.0248	0.0213	-1.1656	0.2439	0.3253
METABRIC	E2F2_plus_MKI67	DNMT1_z	PAM50Luminal B	-0.1013	0.0571	-1.7730	0.0764	0.1223
METABRIC	E2F2_plus_MKI67	DNMT1_z	PAM50HER2-enriched	-0.3992	0.0743	-5.3698	8.938e-08	3.575e-07
METABRIC	E2F2_plus_MKI67	DNMT1_z	PAM50Basal-like	0.3554	0.0864	4.1123	4.100e-05	8.200e-05
METABRIC	E2F2_plus_MKI67	DNMT1_z	PAM50Normal-like	-0.0050	0.0781	-0.0644	0.9487	0.9487
METABRIC	E2F3_plus_MKI67	DNMT1_z	(Intercept)	-0.0681	0.0370	-1.8409	0.0658	0.1053
METABRIC	E2F3_plus_MKI67	DNMT1_z	E2F3_z	0.0893	0.0280	3.1891	0.0015	0.0039
METABRIC	E2F3_plus_MKI67	DNMT1_z	MKI67_z	0.2881	0.0270	10.6568	9.806e-26	7.845e-25
METABRIC	E2F3_plus_MKI67	DNMT1_z	age_z	-0.0190	0.0221	-0.8577	0.3912	0.4471
METABRIC	E2F3_plus_MKI67	DNMT1_z	PAM50Luminal B	0.0679	0.0571	1.1886	0.2348	0.3130
METABRIC	E2F3_plus_MKI67	DNMT1_z	PAM50HER2-enriched	-0.1601	0.0741	-2.1608	0.0308	0.0617
METABRIC	E2F3_plus_MKI67	DNMT1_z	PAM50Basal-like	0.5799	0.0927	6.2568	4.931e-10	1.973e-09
METABRIC	E2F3_plus_MKI67	DNMT1_z	PAM50Normal-like	0.0132	0.0808	0.1638	0.8699	0.8699
METABRIC	E2F_metagene_plus_MKI67	DNMT1_z	(Intercept)	0.1478	0.0385	3.8377	1.286e-04	2.572e-04
METABRIC	E2F_metagene_plus_MKI67	DNMT1_z	E2F_metagene_z	0.5274	0.0377	14.0009	2.705e-42	2.164e-41
METABRIC	E2F_metagene_plus_MKI67	DNMT1_z	MKI67_z	0.0707	0.0302	2.3429	0.0192	0.0308
METABRIC	E2F_metagene_plus_MKI67	DNMT1_z	age_z	0.0011	0.0210	0.0526	0.9580	0.9580
METABRIC	E2F_metagene_plus_MKI67	DNMT1_z	PAM50Luminal B	-0.3476	0.0618	-5.6233	2.177e-08	5.806e-08
METABRIC	E2F_metagene_plus_MKI67	DNMT1_z	PAM50HER2-enriched	-0.4841	0.0738	-6.5622	6.968e-11	2.787e-10
METABRIC	E2F_metagene_plus_MKI67	DNMT1_z	PAM50Basal-like	0.0079	0.0939	0.0843	0.9328	0.9580
METABRIC	E2F_metagene_plus_MKI67	DNMT1_z	PAM50Normal-like	0.0835	0.0770	1.0848	0.2782	0.3709
METABRIC	E2F2_E2F3_joint_plus_MKI67	DNMT1_z	(Intercept)	0.0414	0.0371	1.1161	0.2645	0.3220
METABRIC	E2F2_E2F3_joint_plus_MKI67	DNMT1_z	E2F2_z	0.3477	0.0315	11.0416	1.902e-27	1.712e-26
METABRIC	E2F2_E2F3_joint_plus_MKI67	DNMT1_z	E2F3_z	0.0305	0.0276	1.1032	0.2701	0.3220
METABRIC	E2F2_E2F3_joint_plus_MKI67	DNMT1_z	MKI67_z	0.1381	0.0295	4.6847	3.020e-06	9.060e-06
METABRIC	E2F2_E2F3_joint_plus_MKI67	DNMT1_z	age_z	-0.0228	0.0214	-1.0667	0.2862	0.3220
METABRIC	E2F2_E2F3_joint_plus_MKI67	DNMT1_z	PAM50Luminal B	-0.0973	0.0572	-1.6993	0.0894	0.1610
METABRIC	E2F2_E2F3_joint_plus_MKI67	DNMT1_z	PAM50HER2-enriched	-0.4116	0.0752	-5.4747	5.018e-08	2.258e-07
METABRIC	E2F2_E2F3_joint_plus_MKI67	DNMT1_z	PAM50Basal-like	0.3184	0.0927	3.4347	6.070e-04	0.0014
METABRIC	E2F2_E2F3_joint_plus_MKI67	DNMT1_z	PAM50Normal-like	-0.0060	0.0781	-0.0767	0.9389	0.9389

SUPPLEMENTARY TABLE S1

Full model outputs - confidence intervals and model fit

Part 2 of the full output table; identifier columns are repeated across column blocks.

cohort	model	term	conf low	conf high	n	adj r squared	AIC	BIC	error
TCGA-BRCA	E2F2_plus_MKI67	(Intercept)	0.1173	0.2600	1,067	0.4110	2460.77	2505.53	
TCGA-BRCA	E2F2_plus_MKI67	E2F2_z	0.2049	0.3584	1,067	0.4110	2460.77	2505.53	
TCGA-BRCA	E2F2_plus_MKI67	MKI67_z	0.4177	0.5645	1,067	0.4110	2460.77	2505.53	
TCGA-BRCA	E2F2_plus_MKI67	age_z	-0.0492	0.0442	1,067	0.4110	2460.77	2505.53	
TCGA-BRCA	E2F2_plus_MKI67	PAM50Luminal B	-0.4556	-0.1807	1,067	0.4110	2460.77	2505.53	
TCGA-BRCA	E2F2_plus_MKI67	PAM50HER2-enriched	-0.8250	-0.4435	1,067	0.4110	2460.77	2505.53	
TCGA-BRCA	E2F2_plus_MKI67	PAM50Basal-like	-0.5329	-0.2217	1,067	0.4110	2460.77	2505.53	
TCGA-BRCA	E2F2_plus_MKI67	PAM50Normal-like	-0.3981	0.0951	1,067	0.4110	2460.77	2505.53	
TCGA-BRCA	E2F3_plus_MKI67	(Intercept)	0.0978	0.2307	1,067	0.4622	2363.74	2408.49	
TCGA-BRCA	E2F3_plus_MKI67	E2F3_z	0.3182	0.4361	1,067	0.4622	2363.74	2408.49	
TCGA-BRCA	E2F3_plus_MKI67	MKI67_z	0.4266	0.5476	1,067	0.4622	2363.74	2408.49	
TCGA-BRCA	E2F3_plus_MKI67	age_z	-0.0297	0.0599	1,067	0.4622	2363.74	2408.49	
TCGA-BRCA	E2F3_plus_MKI67	PAM50Luminal B	-0.3023	-0.0432	1,067	0.4622	2363.74	2408.49	
TCGA-BRCA	E2F3_plus_MKI67	PAM50HER2-enriched	-0.6873	-0.3256	1,067	0.4622	2363.74	2408.49	
TCGA-BRCA	E2F3_plus_MKI67	PAM50Basal-like	-0.6211	-0.3287	1,067	0.4622	2363.74	2408.49	
TCGA-BRCA	E2F3_plus_MKI67	PAM50Normal-like	-0.3408	0.1299	1,067	0.4622	2363.74	2408.49	
TCGA-BRCA	E2F_metagene_plus_MKI67	(Intercept)	0.2015	0.3239	1,067	0.5602	2149.16	2193.91	
TCGA-BRCA	E2F_metagene_plus_MKI67	E2F_metagene_z	0.8860	1.0715	1,067	0.5602	2149.16	2193.91	
TCGA-BRCA	E2F_metagene_plus_MKI67	MKI67_z	-0.1889	-0.0142	1,067	0.5602	2149.16	2193.91	
TCGA-BRCA	E2F_metagene_plus_MKI67	age_z	-0.0363	0.0443	1,067	0.5602	2149.16	2193.91	
TCGA-BRCA	E2F_metagene_plus_MKI67	PAM50Luminal B	-0.6865	-0.4442	1,067	0.5602	2149.16	2193.91	
TCGA-BRCA	E2F_metagene_plus_MKI67	PAM50HER2-enriched	-0.7285	-0.4016	1,067	0.5602	2149.16	2193.91	
TCGA-BRCA	E2F_metagene_plus_MKI67	PAM50Basal-like	-0.7277	-0.4652	1,067	0.5602	2149.16	2193.91	
TCGA-BRCA	E2F_metagene_plus_MKI67	PAM50Normal-like	-0.1608	0.2659	1,067	0.5602	2149.16	2193.91	
TCGA-BRCA	E2F2_E2F3_joint_plus_MKI67	(Intercept)	0.1621	0.2953	1,067	0.4912	2305.54	2355.27	
TCGA-BRCA	E2F2_E2F3_joint_plus_MKI67	E2F2_z	0.2135	0.3562	1,067	0.4912	2305.54	2355.27	
TCGA-BRCA	E2F2_E2F3_joint_plus_MKI67	E2F3_z	0.3214	0.4361	1,067	0.4912	2305.54	2355.27	
TCGA-BRCA	E2F2_E2F3_joint_plus_MKI67	MKI67_z	0.2379	0.3848	1,067	0.4912	2305.54	2355.27	
TCGA-BRCA	E2F2_E2F3_joint_plus_MKI67	age_z	-0.0182	0.0690	1,067	0.4912	2305.54	2355.27	
TCGA-BRCA	E2F2_E2F3_joint_plus_MKI67	PAM50Luminal B	-0.3904	-0.1344	1,067	0.4912	2305.54	2355.27	
TCGA-BRCA	E2F2_E2F3_joint_plus_MKI67	PAM50HER2-enriched	-0.7752	-0.4204	1,067	0.4912	2305.54	2355.27	
TCGA-BRCA	E2F2_E2F3_joint_plus_MKI67	PAM50Basal-like	-0.8449	-0.5403	1,067	0.4912	2305.54	2355.27	
TCGA-BRCA	E2F2_E2F3_joint_plus_MKI67	PAM50Normal-like	-0.3797	0.0786	1,067	0.4912	2305.54	2355.27	

SUPPLEMENTARY TABLE S1

Full model outputs - confidence intervals and model fit (continued)

Part 2 of the full output table; identifier columns are repeated across column blocks.

cohort	model	term	conf low	conf high	n	adj r squared	AIC	BIC	error
METABRIC	E2F2_plus_MKI67	(Intercept)	-0.0358	0.1087	1,756	0.2670	4447.76	4497.00	
METABRIC	E2F2_plus_MKI67	E2F2_z	0.2938	0.4150	1,756	0.2670	4447.76	4497.00	
METABRIC	E2F2_plus_MKI67	MKI67_z	0.0854	0.1999	1,756	0.2670	4447.76	4497.00	
METABRIC	E2F2_plus_MKI67	age_z	-0.0666	0.0169	1,756	0.2670	4447.76	4497.00	
METABRIC	E2F2_plus_MKI67	PAM50Luminal B	-0.2133	0.0108	1,756	0.2670	4447.76	4497.00	
METABRIC	E2F2_plus_MKI67	PAM50HER2-enriched	-0.5451	-0.2534	1,756	0.2670	4447.76	4497.00	
METABRIC	E2F2_plus_MKI67	PAM50Basal-like	0.1859	0.5250	1,756	0.2670	4447.76	4497.00	
METABRIC	E2F2_plus_MKI67	PAM50Normal-like	-0.1583	0.1482	1,756	0.2670	4447.76	4497.00	
METABRIC	E2F3_plus_MKI67	(Intercept)	-0.1406	0.0045	1,756	0.2164	4564.99	4614.23	
METABRIC	E2F3_plus_MKI67	E2F3_z	0.0344	0.1442	1,756	0.2164	4564.99	4614.23	
METABRIC	E2F3_plus_MKI67	MKI67_z	0.2351	0.3412	1,756	0.2164	4564.99	4614.23	
METABRIC	E2F3_plus_MKI67	age_z	-0.0623	0.0244	1,756	0.2164	4564.99	4614.23	
METABRIC	E2F3_plus_MKI67	PAM50Luminal B	-0.0441	0.1799	1,756	0.2164	4564.99	4614.23	
METABRIC	E2F3_plus_MKI67	PAM50HER2-enriched	-0.3054	-0.0148	1,756	0.2164	4564.99	4614.23	
METABRIC	E2F3_plus_MKI67	PAM50Basal-like	0.3981	0.7617	1,756	0.2164	4564.99	4614.23	
METABRIC	E2F3_plus_MKI67	PAM50Normal-like	-0.1452	0.1717	1,756	0.2164	4564.99	4614.23	
METABRIC	E2F_metagene_plus_MKI67	(Intercept)	0.0723	0.2233	1,756	0.2914	4388.54	4437.77	
METABRIC	E2F_metagene_plus_MKI67	E2F_metagene_z	0.4535	0.6013	1,756	0.2914	4388.54	4437.77	
METABRIC	E2F_metagene_plus_MKI67	MKI67_z	0.0115	0.1298	1,756	0.2914	4388.54	4437.77	
METABRIC	E2F_metagene_plus_MKI67	age_z	-0.0401	0.0423	1,756	0.2914	4388.54	4437.77	
METABRIC	E2F_metagene_plus_MKI67	PAM50Luminal B	-0.4688	-0.2263	1,756	0.2914	4388.54	4437.77	
METABRIC	E2F_metagene_plus_MKI67	PAM50HER2-enriched	-0.6288	-0.3394	1,756	0.2914	4388.54	4437.77	
METABRIC	E2F_metagene_plus_MKI67	PAM50Basal-like	-0.1762	0.1920	1,756	0.2914	4388.54	4437.77	
METABRIC	E2F_metagene_plus_MKI67	PAM50Normal-like	-0.0675	0.2344	1,756	0.2914	4388.54	4437.77	
METABRIC	E2F2_E2F3_joint_plus_MKI67	(Intercept)	-0.0314	0.1142	1,756	0.2671	4448.54	4503.24	
METABRIC	E2F2_E2F3_joint_plus_MKI67	E2F2_z	0.2859	0.4094	1,756	0.2671	4448.54	4503.24	
METABRIC	E2F2_E2F3_joint_plus_MKI67	E2F3_z	-0.0237	0.0846	1,756	0.2671	4448.54	4503.24	
METABRIC	E2F2_E2F3_joint_plus_MKI67	MKI67_z	0.0803	0.1959	1,756	0.2671	4448.54	4503.24	
METABRIC	E2F2_E2F3_joint_plus_MKI67	age_z	-0.0647	0.0191	1,756	0.2671	4448.54	4503.24	
METABRIC	E2F2_E2F3_joint_plus_MKI67	PAM50Luminal B	-0.2095	0.0150	1,756	0.2671	4448.54	4503.24	
METABRIC	E2F2_E2F3_joint_plus_MKI67	PAM50HER2-enriched	-0.5591	-0.2642	1,756	0.2671	4448.54	4503.24	
METABRIC	E2F2_E2F3_joint_plus_MKI67	PAM50Basal-like	0.1366	0.5003	1,756	0.2671	4448.54	4503.24	
METABRIC	E2F2_E2F3_joint_plus_MKI67	PAM50Normal-like	-0.1593	0.1473	1,756	0.2671	4448.54	4503.24	

SUPPLEMENTARY TABLE S2

Definitions and analysis notes

Linear regression models were fitted with standardized DNMT1 expression as the outcome and standardized E2F2 expression, PAM50 subtype, age, and the E2F2 × PAM50 subtype interaction term as predictors. Luminal A was used as the reference subtype in both cohorts. The table provides subtype-specific E2F2 slope estimates, interaction contrast estimates relative to Luminal A, standard errors, 95% confidence intervals, nominal P values, Benjamini–Hochberg adjusted q values, global likelihood-ratio test statistics comparing models with and without the E2F2 × PAM50 subtype interaction term, model fit indices, and sample sizes. These analyses evaluate subtype-dependent heterogeneity in transcriptomic association and do not establish subtype-specific regulatory mechanisms.

Field	Description
Purpose	This supplementary table provides detailed subtype-specific E2F2-DNMT1 interaction model outputs.
Outcome	Standardized DNMT1 expression.
Predictor	Standardized E2F2 expression.
Model	Linear model: DNMT1_z ~ E2F2_z * PAM50_subtype + age.
Reference subtype	Luminal A
Subtype-specific E2F2 slope	Estimated E2F2-DNMT1 slope within each PAM50 subtype.
Interaction contrast vs reference	Difference in E2F2-DNMT1 slope relative to the reference subtype.
Global likelihood-ratio test	Likelihood-ratio test comparing the main-effects model with the E2F2 x PAM50 interaction model.
P values	Nominal P values are provided for subtype-specific slopes and interaction contrasts.
q values	Benjamini-Hochberg adjusted q values are calculated within each cohort and estimate type.
Covariates	Age and PAM50 subtype annotation.
Interpretation	These analyses evaluate subtype-dependent heterogeneity in transcriptomic association and do not establish subtype-specific regulatory mechanisms.
Confidence intervals	Nominal (unadjusted) two-sided 95% confidence intervals are provided for subtype-specific slopes and interaction contrasts.

SUPPLEMENTARY TABLE S2

Global likelihood-ratio test summary

Likelihood-ratio tests compare models with and without the E2F2 x PAM50 subtype interaction term.

cohort	reference subtype	model main	model interaction	global LRT chisq	global LRT df	global LRT p	n
TCGA-BRCA	Luminal A	DNMT1_z ~ E2F2_z + PAM50_subtype + age	DNMT1_z ~ E2F2_z * PAM50_subtype + age	15.6579	4	0.0035	1,067
METABRIC	Luminal A	DNMT1_z ~ E2F2_z + PAM50_subtype + age	DNMT1_z ~ E2F2_z * PAM50_subtype + age	40.6770	4	3.135e-08	1,756

SUPPLEMENTARY TABLE S2

Model fit summary

Adjusted R-squared, AIC, and BIC values are reproduced from the supplied workbook.

cohort	model type	formula	n	adjusted R2	AIC	BIC
TCGA-BRCA	main effects	DNMT1_z ~ E2F2_z + PAM50_subtype + age	1,067	0.3158	2619.57	2659.35
TCGA-BRCA	E2F2 x PAM50 interaction	DNMT1_z ~ E2F2_z * PAM50_subtype + age	1,067	0.3232	2611.91	2671.58
METABRIC	main effects	DNMT1_z ~ E2F2_z + PAM50_subtype + age	1,756	0.2574	4469.60	4513.37
METABRIC	E2F2 x PAM50 interaction	DNMT1_z ~ E2F2_z * PAM50_subtype + age	1,756	0.2728	4436.92	4502.57

SUPPLEMENTARY TABLE S2

Subtype-specific outputs - estimates and confidence intervals

Part 1 of the subtype-specific E2F2 output table.

cohort	subtype	estimate type	contrast label	estimate	standard error	ci lower	ci upper
TCGA-BRCA	Luminal A	subtype-specific E2F2 slope		0.5237	0.0490	0.4275	0.6199
TCGA-BRCA	Luminal B	subtype-specific E2F2 slope		0.7382	0.0774	0.5864	0.8901
TCGA-BRCA	HER2-enriched	subtype-specific E2F2 slope		0.8893	0.1409	0.6129	1.1658
TCGA-BRCA	Basal-like	subtype-specific E2F2 slope		0.6633	0.0579	0.5497	0.7770
TCGA-BRCA	Normal-like	subtype-specific E2F2 slope		0.2745	0.1506	-0.0210	0.5699
TCGA-BRCA	Luminal B	interaction contrast vs reference	Luminal B - Luminal A	0.2145	0.0913	0.0354	0.3936
TCGA-BRCA	HER2-enriched	interaction contrast vs reference	(HER2-enriched) - Luminal A	0.3656	0.1494	0.0725	0.6587
TCGA-BRCA	Basal-like	interaction contrast vs reference	(Basal-like) - Luminal A	0.1396	0.0757	-0.0089	0.2882
TCGA-BRCA	Normal-like	interaction contrast vs reference	(Normal-like) - Luminal A	-0.2492	0.1582	-0.5597	0.0612
METABRIC	Luminal A	subtype-specific E2F2 slope		0.3479	0.0476	0.2545	0.4412
METABRIC	Luminal B	subtype-specific E2F2 slope		0.7182	0.0526	0.6150	0.8215
METABRIC	HER2-enriched	subtype-specific E2F2 slope		0.2785	0.0725	0.1364	0.4207
METABRIC	Basal-like	subtype-specific E2F2 slope		0.3340	0.0596	0.2170	0.4510
METABRIC	Normal-like	subtype-specific E2F2 slope		0.3472	0.0848	0.1809	0.5135
METABRIC	Luminal B	interaction contrast vs reference	Luminal B - Luminal A	0.3704	0.0710	0.2312	0.5095
METABRIC	HER2-enriched	interaction contrast vs reference	(HER2-enriched) - Luminal A	-0.0693	0.0867	-0.2394	0.1007
METABRIC	Basal-like	interaction contrast vs reference	(Basal-like) - Luminal A	-0.0139	0.0763	-0.1635	0.1358
METABRIC	Normal-like	interaction contrast vs reference	(Normal-like) - Luminal A	-6.490e-04	0.0972	-0.1914	0.1901

SUPPLEMENTARY TABLE S2

Subtype-specific outputs - significance

Part 2 of the subtype-specific output table; identifier columns are repeated for readability.

cohort	subtype	estimate type	df	statistic	p value	q value	n
TCGA-BRCA	Luminal A	subtype-specific E2F2 slope	1,056	10.6805	2.344e-25	5.861e-25	1,067
TCGA-BRCA	Luminal B	subtype-specific E2F2 slope	1,056	9.5404	9.484e-21	1.581e-20	1,067
TCGA-BRCA	HER2-enriched	subtype-specific E2F2 slope	1,056	6.3125	4.035e-10	5.043e-10	1,067
TCGA-BRCA	Basal-like	subtype-specific E2F2 slope	1,056	11.4515	1.055e-28	5.273e-28	1,067
TCGA-BRCA	Normal-like	subtype-specific E2F2 slope	1,056	1.8226	0.0686	0.0686	1,067
TCGA-BRCA	Luminal B	interaction contrast vs reference	1,056	2.3502	0.0189	0.0379	1,067
TCGA-BRCA	HER2-enriched	interaction contrast vs reference	1,056	2.4477	0.0145	0.0379	1,067
TCGA-BRCA	Basal-like	interaction contrast vs reference	1,056	1.8443	0.0654	0.0872	1,067
TCGA-BRCA	Normal-like	interaction contrast vs reference	1,056	-1.5754	0.1155	0.1155	1,067
METABRIC	Luminal A	subtype-specific E2F2 slope	1,745	7.3098	4.055e-13	1.014e-12	1,756
METABRIC	Luminal B	subtype-specific E2F2 slope	1,745	13.6459	2.327e-40	1.164e-39	1,756
METABRIC	HER2-enriched	subtype-specific E2F2 slope	1,745	3.8436	1.256e-04	1.256e-04	1,756
METABRIC	Basal-like	subtype-specific E2F2 slope	1,745	5.5994	2.495e-08	4.158e-08	1,756
METABRIC	Normal-like	subtype-specific E2F2 slope	1,745	4.0955	4.404e-05	5.505e-05	1,756
METABRIC	Luminal B	interaction contrast vs reference	1,745	5.2199	2.004e-07	8.016e-07	1,756
METABRIC	HER2-enriched	interaction contrast vs reference	1,745	-0.7999	0.4239	0.8477	1,756
METABRIC	Basal-like	interaction contrast vs reference	1,745	-0.1819	0.8557	0.9947	1,756
METABRIC	Normal-like	interaction contrast vs reference	1,745	-0.0067	0.9947	0.9947	1,756

SUPPLEMENTARY TABLE S2

Subtype-specific outputs - model details

Part 3 of the subtype-specific output table, including the global interaction test and model specification.

cohort	subtype	subtype annotation	reference subtype	global LRT chisq	global LRT df	global LRT p	model	covariates
TCGA-BRCA	Luminal A	PAM50_subtype	Luminal A	15.6579	4	0.0035	DNMT1_z ~ E2F2_z * PAM50_subtype + age	age + PAM50_subtype
TCGA-BRCA	Luminal B	PAM50_subtype	Luminal A	15.6579	4	0.0035	DNMT1_z ~ E2F2_z * PAM50_subtype + age	age + PAM50_subtype
TCGA-BRCA	HER2-enriched	PAM50_subtype	Luminal A	15.6579	4	0.0035	DNMT1_z ~ E2F2_z * PAM50_subtype + age	age + PAM50_subtype
TCGA-BRCA	Basal-like	PAM50_subtype	Luminal A	15.6579	4	0.0035	DNMT1_z ~ E2F2_z * PAM50_subtype + age	age + PAM50_subtype
TCGA-BRCA	Normal-like	PAM50_subtype	Luminal A	15.6579	4	0.0035	DNMT1_z ~ E2F2_z * PAM50_subtype + age	age + PAM50_subtype
TCGA-BRCA	Luminal B	PAM50_subtype	Luminal A	15.6579	4	0.0035	DNMT1_z ~ E2F2_z * PAM50_subtype + age	age + PAM50_subtype
TCGA-BRCA	HER2-enriched	PAM50_subtype	Luminal A	15.6579	4	0.0035	DNMT1_z ~ E2F2_z * PAM50_subtype + age	age + PAM50_subtype
TCGA-BRCA	Basal-like	PAM50_subtype	Luminal A	15.6579	4	0.0035	DNMT1_z ~ E2F2_z * PAM50_subtype + age	age + PAM50_subtype
TCGA-BRCA	Normal-like	PAM50_subtype	Luminal A	15.6579	4	0.0035	DNMT1_z ~ E2F2_z * PAM50_subtype + age	age + PAM50_subtype
METABRIC	Luminal A	PAM50_subtype	Luminal A	40.6770	4	3.135e-08	DNMT1_z ~ E2F2_z * PAM50_subtype + age	age + PAM50_subtype
METABRIC	Luminal B	PAM50_subtype	Luminal A	40.6770	4	3.135e-08	DNMT1_z ~ E2F2_z * PAM50_subtype + age	age + PAM50_subtype
METABRIC	HER2-enriched	PAM50_subtype	Luminal A	40.6770	4	3.135e-08	DNMT1_z ~ E2F2_z * PAM50_subtype + age	age + PAM50_subtype
METABRIC	Basal-like	PAM50_subtype	Luminal A	40.6770	4	3.135e-08	DNMT1_z ~ E2F2_z * PAM50_subtype + age	age + PAM50_subtype
METABRIC	Normal-like	PAM50_subtype	Luminal A	40.6770	4	3.135e-08	DNMT1_z ~ E2F2_z * PAM50_subtype + age	age + PAM50_subtype
METABRIC	Luminal B	PAM50_subtype	Luminal A	40.6770	4	3.135e-08	DNMT1_z ~ E2F2_z * PAM50_subtype + age	age + PAM50_subtype
METABRIC	HER2-enriched	PAM50_subtype	Luminal A	40.6770	4	3.135e-08	DNMT1_z ~ E2F2_z * PAM50_subtype + age	age + PAM50_subtype
METABRIC	Basal-like	PAM50_subtype	Luminal A	40.6770	4	3.135e-08	DNMT1_z ~ E2F2_z * PAM50_subtype + age	age + PAM50_subtype
METABRIC	Normal-like	PAM50_subtype	Luminal A	40.6770	4	3.135e-08	DNMT1_z ~ E2F2_z * PAM50_subtype + age	age + PAM50_subtype
